# Supplementary material for: Global implications of uncertainty in China’s climate policy delivery
Source: Nat Commun. 2026 Mar 6;17:3544. doi: 10.1038/s41467-026-70400-8 (PMC13087136; doi:10.1038/s41467-026-70400-8)
Supplement: Supplementary file 1 — Supplementary Information [file 41467_2026_70400_MOESM1_ESM.pdf]

Global implications of uncertainty in China's climate policy delivery: supplementary information

Table of Contents

SI Section 1: Policy credibility assessment of China climate policies 2
Step 1: Identifying the scope of climate policies and their associated targets 5
Step 2: Policy filtering and progress track 7
Step 3: Estimating credibility ratings across different policy targets 9
SI Section 2: Morphological analysis of China's energy transformation 32
SI Section 3 : China energy transformation scenarios 35
Green Lights Scenario 35
Red Sun Scenario 36
Calm Sea Scenario 38
Great Wall Scenario 39
SI Section 4: Scenario quantification and key assumptions 40
4.1 National Climate policy delivery 40
4.2 Economic growth 43
4.3 Population changes 45
4.4 Clean technology deployment 47
4.5 CCS deployment 49
SI Section 5: Energy Service Demand in TIAM-UCL 50
SI Section 6: Sensitivity analysis 52
6.1 Credibility score weighting 52
6.2 China's GHG emissions reduction target by 2035 54
Reference 57

SI Section 1: Policy credibility assessment of China climate policies

When seeking to explore future energy scenarios, policy credibility has started to receive attention in the context of achieving a low-carbon transition and meeting climate targets [1–5]. Existing Chinese policy-related scenarios studies have mainly focused on overarching NDC or Net zero targets, often assuming the delivery of policy targets while overlooking the uncertainties associated with delayed, partial, or failed implementation—particularly at the sectoral level. This paper takes a first step toward closing that research gap by systematically assessing the credibility of China's sectoral climate and energy targets, providing a more realistic basis for evaluating whether the country is on track to meet its decarbonisation goals.

The definition of 'policy credibility' varies in the literature [6–8]. Furthermore, there is no standard assessment framework for policy credibility across studies[4,5]. In this study, we follow Rogelj, J. *et al* [3], who define credibility as "the level of confidence in a target (net-zero targets) delivery", and extend their net-zero target credibility assessment to national-level targets in China. Policies reviewed in this study encompass all Chinese government policy documents related to energy and climate issued from central government between 2020 and 2025, including action plans, working scheme, strategy documents, guiding opinions, guidelines, standards and so on. The credibility rating in Rogelj et al.'s study is based on three criteria: (i) whether a country's net-zero target is legally binding, (ii) whether it is accompanied by a published implementation plan, and (iii) whether the country's current policy trajectory is on track to significantly reduce emissions by 2030. In the context of China, we conducted credibility rating based on the following three policy characteristics:

Governance level at which policy is set: we replace the "legally binding" criterion with the governance level at which the policy is set as a more nuanced and accurate criteria of credibility in the Chinese context. China's governance system is hierarchical and predominantly driven by executive orders rather than acts of parliament[9,10]. Legislation and policies related to climate change are often issued as soft regulations, rather than hard laws[11], which shifts the focus from legal enforceability to the authority and level of governance behind a policy[12,13] (see SI Figure 1) . The "dual-carbon" targets (peaking carbon emissions by 2030 and achieving carbon neutrality by 2060) are an example of policies set at the highest levels of authority. These targets are endorsed by the Chinese Communist Party (CCP) and the State Council and are made legally binding at the national level. In contrast, policies issued by the National Development and Reform Commission (NDRC) or other ministries are often non-binding guidelines or frameworks. Therefore, to better evaluate the credibility of all official targets from all levels of

government, it cannot be assessed simply using the "legally binding" as is common in other countries. Instead, the level of authority behind the policy—whether it is set by the CCP, the State Council, the NDRC, or ministries—plays a significant role in determining its enforceability and alignment with the overarching dual-carbon goals. Policies issued by higher authorities, such as the CCP and the State Council, typically receive stronger institutional support, better funding, and more rigorous monitoring, marking them more likely to secure compliance and achieve their intended outcomes[14]. In contrast, lower-level policies often depend on the interpretation and implementation capacity of subnational governments, which can result in inconsistencies and gaps in execution[15]. Therefore, we assume the achievement of the climate target with higher-level endorsement and multiple reaffirmations from top to down is more credible.

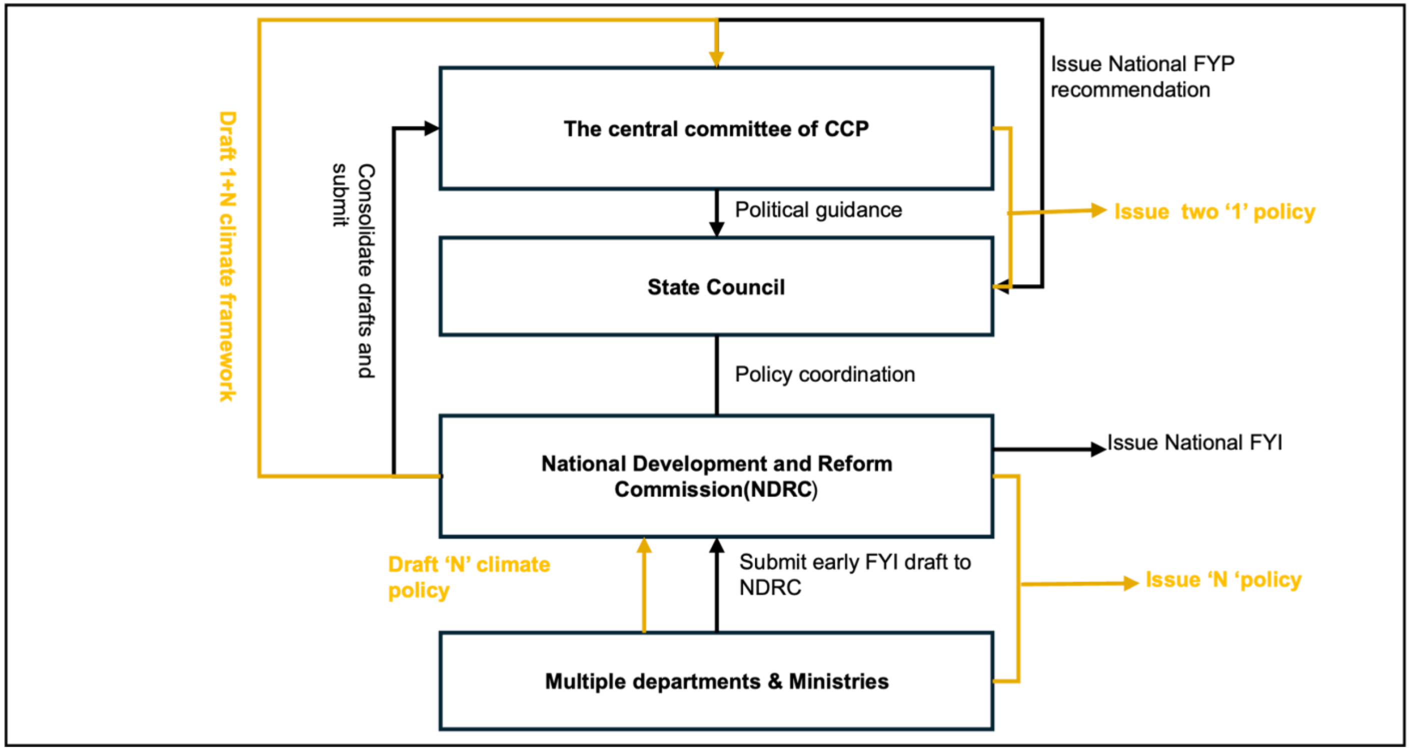

SI Figure 1.Overview of China's climate governance framework led by the central government adapted based on Teng and Wang[12], Li et al.[16]

Clearly stated in a five-year plan:

The 14th Five-Year Plan (FYP) (2020–2025) plays a pivotal role in China's climate governance by setting clear targets, defining strategic initiatives, and embedding environmental objectives into the nation's overall development agenda. By integrating climate objectives into the broader economic planning framework, the FYP ensures that climate governance aligns with the country's development priorities, fosters coordinated action across various levels of government and sectors and outlines specific strategies for achieving the dual-carbon targets, such as promoting renewable energy and advancing low-carbon technologies[16]. Moreover, the targets set in the 14th FYP are typically cautious and realistic, closely tied to government performance evaluations. This connection enhances their executive nature, making them more actionable and subject to evaluation at the end of the five-year period[17]. Such evaluation and report provide mechanisms for accountability and progress monitoring. Therefore, we assume that climate targets clearly stated in a Five-Year Plan are more actionable and credible due to their integration into performance evaluations, alignment with broader development priorities, and a pragmatic approach to implementation.

Time normalised progress index (TNPI): With China's 14th Five-Year Plan nearing completion, evaluating the extent to which each target is on track to be achieved will provide a foundation for developing new policies, plans, and targets for the next five years. Given the uncertainties in tracking broad goals like carbon neutrality and peak emissions, as well as the need for further model quantification, our assessment focuses exclusively on quantifiable, trackable, and statistically measurable targets (see SI Table 3). All evidence sources on progress are provided in the supplementary data.

For each target, we first review and calculate raw progress as the share of the indicator gap closed since the base year, as equation (1) shows:

$$\boldsymbol{Raw\ progress = \frac{C_v - B_v}{T_v - B_v}} \qquad \textbf{(1)}$$

Where B_v , T_v, C_v are the base, target, and current values of the indicator. Targets where raw progress ≥100% are classified as ***Achieved*.** To ensure comparability across targets with different deadlines, we assume policy indicators advance along a linear trajectory and normalise progress by the proportion of time elapsed. This allows both short-and long-term goals to be evaluated based on whether progress is keeping on track with time passed. The resulting time-normalised progress index (TNPI) is given by equation (2):

$$\boldsymbol{TNPI = \frac{(C_v - B_v)/(T_v - B_v)}{(C_y - B_y)/(T_y - B_y)} * 100\%} \qquad \textbf{(2)}$$

Where B_y , T_y, C_y are the base, target, and current years. TNPI ≥100% indicate the target is on or ahead of schedule; TNPI <100% indicate it is lagging. For infrastructure-related targets with long lead times (e.g., nuclear power, hydropower), we use operational and under-construction capacity as the current value to better account for construction lead times.

For targets still in progress (raw progress < 1), we classify them using a **weighted credibility score**. Each target's score combines three components: governance level, inclusion in the 14th Five-Year Plan, and the TNPI. To ensure robustness, we tested five alternative weighting schemes (See SI section 6.1). Targets are then clustered into categories as follows: ***High credible*** (score ≥ 67th percentile), ***Medium credible*** (33rd–67th percentile), and ***Low credible*** (below 33rd percentile). Comparing clustering results across weighting schemes, target classifications are largely robust. Only two targets shift category when the weight on TNPI is smallest ($\omega_{gov}\colon \omega_{FYP}\colon \omega_{TNPI} = 4\colon 4\colon 2$). We therefore adopt the first four weighting schemes as our main case and report additional sensitivity results in SI Section 6.1.

Step 1: Identifying the scope of climate policies and their associated targets

Climate change as a key issue is not only reflected in the NDC and net zero targets but also in the recent five-year plans and other high-level government documents involving energy and emissions governance. The policies reviewed in this study are those issued from 2020 to September 2025 and include:

- **China's updated NDC 2035:** At the United Nations General Assembly on 25 September 2025**,** President Xi announced China's new 2035 NDC targets in a video address, outlining three core commitments:
    - Reduce economy-wide net GHG emissions by 7% to 10% below peak levels by 2035.
    - Increase the share of non-fossil energy in total energy consumption to over 30% by 2035.
    - Expand the installed capacity of wind and solar to 3,600 GW by 2035.

  Drawing on past experience, such as the 2021 carbon neutrality pledge, commitments made by the top leadership are typically incorporated into China's overarching policy framework and subsequently embedded in sectoral planning and regulatory measures. As a result, the 2035 NDC announcement is likely to shape the trajectory of China's long-term decarbonization strategy, providing an important signal of the country's intended direction. Its implementation will therefore have significant implications for China's emissions pathway

and, by extension, for global climate outcomes. The targets on non-fossil energy share and wind and solar installed capacity are extensions of existing pledges. We integrated these into our policy credibility assessment framework. However, the GHG emissions target is China's first explicit absolute emissions reduction pledge. As there is no historical progress against which to benchmark it, we treat this as an additional policy sensitivity to our main case (see SI section 6.2).

- **Policies within the "1+N" climate policy framework:** The "1+N" climate policy framework is a strategic plan introduced to achieve its goals of carbon peak before 2030 and carbon neutrality before 2060. The "1" refers to the guiding document or the top-level policy guideline that sets the overarching goals and principles for China's climate actions. The "N" represents a series of specific action plans and measures that will be implemented across different sectors and regions to achieve the goals outlined in the "1" document. **T**he "1" policy *The Action Plan for Carbon Peaking Before 2030* explicitly proposes 10 actions to achieve a carbon peak by 2030 (see SI Table 1). However, within the scope of "N", there is no official definition of the policies associated with the 10 actions. Therefore, this analysis starts with a comprehensive review of China's climate related policies, according to the 1+N climate policy framework. We dive into the N policies to identify what government actions belong to this scope. Improving the policy transparency and enhancing the understanding the climate efforts from Chinese government is another contribution from our assessment.

SI Table 1.The "1+N" climate policy framework

**Carbon peak before 2030 and carbon net zero before 2060**	
"1": Top-level working guidance	
Working Guidance for Carbon Dioxide Peaking and Carbon Neutrality in Full and Faithful Implementation of the New Development Philosophy	The Action Plan for Carbon Peaking Before 2030
"N": underpinning 10 key actions	

The action for green and low-carbon energy transition	The action for promoting circular economy in carbon mitigation purpose
The action for energy saving carbon emission mitigation and efficiency improvement	The action for advancing green and low-carbon technology innovation
The action for peaking carbon dioxide emissions in industry sector	The action for consolidating and enhancing carbon sink
The action for peaking carbon dioxide emissions in urban-rural development area	The action for green and low-carbon society
The action for promoting green and low-carbon transportation	The action for promoting all regions to peak carbon dioxide emissions hierarchically and orderly

- **Energy and emission policies within 14th Five-Year Plan:** The 14th Five-Year Plan refers to the national development strategy and economic plan covering the years 2021 to 2025, focusing on economy growth, technological innovation, and sustainability. Since China submitted its NDC target, green and low-carbon development has also been incorporated into the overall plans for national economic and social development [18]. Some of the policies in national five-year plan are also supportive of '1+N' climate policy. Therefore, we integrated them into the subcategory of 1+N framework.
- **Ad-hoc energy policies, planning, and standards for different sectors**, including buildings, transport, power, and industry. To ensure comprehensive coverage, we also reviewed the IEA policy dataset and Climate action tracker (CAT) and integrated them into the ten actions under 1+N climate policy framework

Step 2: Policy filtering and progress track

After reviewing all relevant policies in step 1, we collected 58 policy documents and recorded all numerical targets to ensure full coverage. In total, we identified 292 targets (including duplicates, which enabled us to track the frequency with which each target was referenced across policies). To assess model applicability, each target was classified according to whether it was quantifiable in TIAM-UCL. We filtered the targets based on our modelling

expertise and documented the reasons for exclusion for transparency. The reasons for exclusion and illustrative examples are presented in SI Table 2, while the full list is available in supplementary data.

SI Table 2.Rationale for filtering TIAM-quantifiable targets

**Rationale for filtering TIAM-quantifiable targets**	Summary of Reasons	Examples of Targets
**1. Technology / Commodity Not Represented**	TIAM models technologies in aggregate but cannot capture disaggregated forms, infrastructure length, or building floor area. Similarly, some industrial commodities, such as nonferrous metals, are represented in aggregate, preventing explicit representation of specific sectors like aluminium and cement.	• By 2025, the national oil and gas pipeline network will expand to approximately 210,000 km.<br>• By 2025, the energy consumption per unit of cement clinker will be reduced by 3.7%.<br>• By 2025, carbon emissions in the electrolytic aluminium sector will decrease by 5%.
**2. Water, Waste, Recycling, and Non-Energy Resource Flows**	Water use, recycling, waste treatment, secondary materials, and industrial by-products are outside TIAM's scope. The model does not track paper, metals, slag, gypsum, manure, straw, red mud, or appliance/vehicle recycling, which are actually reflected in policies	• By 2025, reduce water use per RMB 10,000 of industrial added value by 16% compared to 2020.<br>• By 2025, achieve a nationwide water reuse rate of approximately 94% in large-scale industrial sectors.
**3. Pollution, Non-GHG Metrics**	TIAM cannot represent non-GHG pollutants, this includes PM2.5, COD, NOx, VOCs.	• By 2025, total emissions of COD, ammonia nitrogen, NOx, and VOCs will decrease by 8%, 8%, >10%, and >10% respectively compared to 2020.<br>• By 2025, operational ships will achieve a 7% reduction in NOx emissions compared to 2020 levels.
**4. Industrial Standards & Economic / Financial Indicators**	TIAM does not model regulatory compliance, benchmark standards, retrofits, capacity ceilings, or process ratios. It also does not track R&D spending, or financial/economic targets.	• Since 2021, establish RMB 200 billion special refinancing loan to support the clean and efficient use of coal.<br>• By 2025, increase the digitalization rate of production equipment to 55%.

**5. Non-measurable / Ambiguous Targets**	Vague target statements lacking clear definitions or involving unmeasurable metrics.	• By 2025, installed capacity of new types of energy storage will reach 30 GW or more.<br>• By 2025, renovation area of existing buildings will increase by >20 million m^2 compared to 2023.<br>• By 2030, all ground vehicles and equipment at civil airports will strive to be powered by electricity.

After filtering, we obtained 90 quantifiable national-level targets (including duplicates), which were consolidated into a final set of 47 targets for the credibility assessment (see SI Table 3, Step 3). As a result, our findings of these study may underestimate the mitigation potential of these measures in GW and RS. Further quantitative research is needed to assess the decarbonisation potential of these targets.

Regarding the impacts of these exclusions, for reasons 1–4, we acknowledge that this TIAM-based study may not fully capture the mitigation effects of the excluded targets. As a result, our findings may underestimate the mitigation potential of these measures in GW and RS. Further quantitative research is needed to assess the decarbonisation potential of these targets. However, IEA data for 2023 show that 96% of China's CO_2 emissions come from energy-related sectors[19], including electricity and heat production, transport, industry, and buildings (residential, commercial, and public services) which are largely covered by our policy assessment (47 quantifiable targets) and TIAM modelling scope. Therefore, the implications of any underestimation arising from the excluded targets (e.g. water, waste, and pollution) for the overall energy transition and China's carbon emissions are limited.

For reason 5, these targets are unlikely to be systematically implemented or tracked in practice. Their exclusion therefore does not alter results but reflect a systemic feature of Chinese climate policymaking, the prevalence of targets that lack measurability or enforceability, provide additional qualitative insight for this paper.

Step 3: Estimating credibility ratings across different policy targets

For each quantifiable target, we extract the base year, base value, target year, and target value, and then review or estimate the most recent progress from public literature. Raw progress is calculated using Equation (1) and the time-normalised progress index (TNPI) using Equation (2). From these, we derive a weighted credibility score and assign each target to a cluster according to the rules outlined above. To account for

the impact of weighting choices on credibility scores, we test five alternative weighting schemes. Table 3 presents one example ($\omega_{gov}:\omega_{FYP}:\omega_{TNPI} = 3:2:5$), while the full credibility assessment sources and processes are provided in supplementary data.

SI Table 3.Overview of China's quantifiable climate targets and the key characteristics underlying the credibility assessment for their realisation

**Target**	**Impacting scope**	**Policy coverage frequency**	**Policy-making authority**	**Current progress (By 2023/2024)**	**Target year**	**Target value**	**Base year**	**Base value**	**Current year**	**Current value**	**Raw progress**	**Time fraction**	**TNPI**	**Governance level**	**Clearly stated in a five-year plan**	**Credibility score (W_gov:W_FYP:W_Prog=3:2:5)**	**Cluster**
By 2025, energy consumption per unit of GDP will be lowered by 13.5% from the 2020 level.	Overall	Working Guidance for Carbon Dioxide Peaking and Carbon Neutrality in Full and Faithful Implementation of the New Development Philosophy<br>The Action Plan for Carbon Peaking Before 2030<br>14th Five-Year Plan on Modern Energy System Planning<br>The "14th Five-Year Plan" Comprehensive Work Plan for Energy Conservation and Emission Reduction<br>14th Five-Year Plan for Circular Economy Development	CPC Central Committee & State council	By 2023, energy intensity has reduced by **7.6%** from 2020 level, based on the data published by National Bureau of Statistics of China; Most recent assessment from Carbon action tracker: progress from 2020-24 was minimal, with only a **5.3%** drop in energy intensity	**2025**	-13.50%	**2020**	0	2024	-5.30%	39.26%	80.00%	**49.07%**	**3**	**2**	**0.74537037**	**MEDIUM**
By 2025, carbon dioxide (CO_2) emissions per unit of GDP will be lowered by	Overall	Working Guidance for Carbon Dioxide Peaking and Carbon Neutrality in Full and Faithful Implementation of the New Development Philosophy	CPC Central Committee &State council	By 2023, carbon intensity has reduced by **6.3%** from 2020 level, based on the data published by National Bureau of Statistics of China<br>By 2024, CREA	**2025**	-18%	**2020**	0	2024	-7.90%	43.89%	80.00%	**54.86%**	**3**	**2**	**0.774305556**	**MEDIUM**

18% from the 2020 level.		The Action Plan for Carbon Peaking Before 2030<br>14th Five-Year Plan on Modern Energy System Planning		estimates a total reduction of 7.9% from 2020 to 2024												
By 2025, the share of non-fossil energy consumption will reach around 20%.	Overall	Working Guidance for Carbon Dioxide Peaking and Carbon Neutrality in Full and Faithful Implementation of the New Development Philosophy<br>The Action Plan for Carbon Peaking Before 2030<br>14th Five-Year Plan on Modern Energy System Planning<br>The "14th Five-Year Plan" Comprehensive Work Plan for Energy Conservation and Emission Reduction<br>2024-2025 Energy Conservation and Carbon Reduction Action Plan	CPC Central Committe e & State council	By 2023, the share of non-fossil fuels in primary energy consumption has increased to 17.5%, according to mid-term evaluation report of the National Development and Reform Commission (15.3% in 2020)<br>By 2024, the proportion of non-fossil energy in primary energy consumption reached 19.7%	**2025**	20%	**2020**	15.30%	2024	19.70%	93.62%	80.00%	**117.02 %**	**3**	**2**	**1.085106383**	**HIGH**
By 2025, the forest coverage rate will reach 24.1%, and the forest stock volume will rise to 18 billion cubic meters.	Overall	Working Guidance for Carbon Dioxide Peaking and Carbon Neutrality in Full and Faithful Implementation of the New Development Philosophy<br>The Action Plan for Carbon Peaking Before	CCP &State Council	China daily reported that the forest coverage rate surpassed 25 percent, while the forest stock volume exceeded 20 billion cubic meters, by 2024	**2025**	18	**2020**	17.56	2024	20	554.55%	80.00%	**693.18 %**	**3**	**2**	**3.965909091**	**ACHIEVED**

		2030														
By 2025, electricity will account for about 30% of end-use energy consumption.	Overall	14th Five-Year Plan on Modern Energy System Planning	NDRC, NEA	Both IEA and Chinese government estimate that around 28% of final energy consumption is electricity by 2023 (27% in 2020) China's electrification rate reached 28.8% last year, an increase of 0.9 percentage points from 2023, already surpassing that of "major developed economies in Europe and the US", China Energy Net says, citing a new report by China Electricity Council.	**2025**	30%	**2020**	27%	2024	29%	60.00%	80.00%	**75.00%**	**2**	**2**	**0.775**	**MEDIUM**
By 2025, total renewable energy consumption will reach about 1 billion tons of standard coal (~29,300 PJ).	Overall	14th Five-Year Plan of Renewable Energy	NDRC	Total renewable energy consumption rose from 0.68 Gtce (~19,930 PJ) in 2020 to ~0.75 Gtce (~21,980 PJ) in 2021 (government achievement reports). By 2023, renewable power generation reached 3,000 TWh (10,800 PJ of electricity). Under the substitution method (assuming	**2025**	29300	**2020**	19930	2023	25500	59.45%	60.00%	**99.08%**	**2**	**2**	**0.895375311**	**MEDIUM**

				40–45% average fossil power efficiency), this corresponds to ~24,000–27,000 PJ of renewable primary energy equivalent.														
By 2025, renewable energy will account for about 18% of primary energy consumption.	Overall	14th Five-Year Plan of Renewable Energy	NDRC	Total renewable energy consumption increased to 14.2% of primary energy consumption in 2022, up from 13.6% in 2020 (as reported by the government). No more recent data are available.	**2025**	18%	**2020**	13.60%	2022	14.20%	13.64%	40.00%	**34.09%**	**2**	**2**	**0.570454545**	**LOW**
By 2025, renewable energy will account for more than 50% of the incremental increase in primary energy consumption.	Overall	14th Five-Year Plan of Renewable Energy	NDRC	No evidence, assume China is approaching this target and by 2023, the progress is 50%	**2025**	50%	**2020**	-	2024	-	50.00%	80.00%	**62.50%**	**2**	**2**	**0.7125**	**MEDIUM**
By 2025, the non-electric utilization of renewable energy (including geothermal heating, biomass heating,	Overall	14th Five-Year Plan of Renewable Energy	NDRC	No evidence, assume China is approaching this target and by 2023, the progress is 50%	**2025**	60	**2020**	-	2024	-	50.00%	80.00%	**62.50%**	**2**	**2**	**0.7125**	**MEDIUM**

biomass fuels, and solar thermal utilization) will reach over 60 million tons of standard coal (~1,758 PJ).																
By 2025, annual crude oil production will stabilize at around 200 million tons.	Upstream	14th Five-Year Plan on Modern Energy System Planning Seven-Year Exploration and Production Increase Action Plans	NDRC	In 2023, **208.91** million tons of crude petroleum oil were produced, according to NBSC.<br>By 2024, S&P estimate 212 million tons of crude petroleum oil were produced	**2025**	200	**2020**	195	2024	212	100.00%	80.00%	**125.00 %**	**2**	**2**	**1.025**	**ACHIEVED**
By 2025, annual natural gas production will exceed 230 billion cubic meters.	Upstream	14th Five-Year Plan on Modern Energy System Planning Seven-Year Exploration and Production Increase Action Plans	NDRC	In 2023, **232** bcm gas were produced domestically, according to NBSC.<br>In 2024, China's total natural-gas output is estimated at 246 billion cubic meters, from Xinhua report	**2025**	230	**2020**	192.5	2024	246	100.00%	80.00%	**125.00 %**	**2**	**2**	**1.025**	**ACHIEVED**
By 2025, 100,000–200,000 tons of hydrogen will be produced annually from renewable energy.	Upstream	Medium- and long-term plan for the development of the hydrogen energy industry (2021-2035)	NDRC	Most hydrogen in China in 2020 was “grey hydrogen” from coal gasification (≈60% of total) and natural gas. China installed a cumulative 1 GW of electrolyser capacity in 2023.Rystad Energy projected the installation of	**2025**	100000-200000	**2020**	0	2024	125000	100.00%	80.00%	**125.00 %**	**2**	**1**	**0.925**	**ACHIEVED**

				approximately 2.5 gigawatts (GW) of hydrogen electrolyser capacity by the end of 2024. As of end-2024, China had established about 125,000 tons/year of green hydrogen production capacity (i.e. electrolytic hydrogen using renewables) according to the National Energy Administration (NEA).													
By 2025, total installed power generation capacity will reach approximately 3,000 GW.	Power sector	14th Five-Year Plan on Modern Energy System Planning	NDRC	By 2024, China's total installed power generation capacity reached 3,349 GW, a year-on-year increase of 14.6%	**2025**	3000	**2020**	2200	2024	3349	143.63%	80.00%	**179.53 %**	**2**	**2**	**1.29765625**	**ACHIEVED**
By 2025, the proportion of non-fossil energy in power generation will reach about 39%.	Power sector	14th Five-Year Plan on Modern Energy System Planning 2024-2025 Energy Conservation and Carbon Reduction Action Plan	NDRC	In 2023, non-fossil share of power generation increased to **34.46%** from 32% in 2020	**2025**	39%	**2020**	32%	2023	34.46%	35.14%	60.00%	**58.57%**	**2**	**2**	**0.692857143**	**MEDIUM**
By 2025, the operational installed capacity of nuclear power will reach	Power sector	14th Five-Year Plan on Modern Energy System Planning	NDRC	According to Chinese government and EIA, the operational installed capacity of nuclear power has reached approximately 53.2	**2025**	70	**2020**	50	2025	76.9	134.50%	100.00%	**134.50 %**	**2**	**2**	**1.0725**	**ACHIEVED**

approximately 70 GW.				GW by 2024 (50GW in 2020)<br>With 53.2 GW already operating and 23.7 GW under construction at end-2024, China has 81 GW either in operation or being built.												
By 2025, power generation from renewable energy will reach approximately 3,300 TWh.	Power sector	14th Five-Year Plan of Renewable Energy	NDRC	By 2024, power generation from renewable energy reach approximately 3460 TWh (2214 TWH in 2020)	**2025**	3300	**2020**	2214	2024	3460	114.73%	80.00%	**143.42 %**	**2**	**2**	**1.117081031**	**ACHIEVED**
By 2025, 33% of electricity will be generated from renewables.	Power sector	14th Five-Year Plan of Renewable Energy	NDRC	The NEA's evaluation report showed renewable electricity consumption totalled 2,444.6 terawatt-hours in 2021, accounting for 29.4% of total electricity consumption. Ember shows China's renewable energy sources generated 35% of the country's electricity in 2023. (28.8% in 2020)<br>In 2024, China generated about 3.46 trillion kWh of electricity from renewable sources,	**2025**	33%	**2020**	28.80%	2024	34%	130.95%	80.00%	**163.69 %**	**2**	**2**	**1.218452381**	**ACHIEVED**

				account for 34.3% of total generation, based on government report												
By 2025, 18% of electricity will come from non-hydropower renewables.	Power sector	14th Five-Year Plan of Renewable Energy	NDRC	In 2023, electricity from non-hydropower renewables has increased to 16.45% (10% in 2020) Combined wind + solar in 2024 ≈ 1,825.7 TWh (or 1.83 trillion kWh) based on NEA data., the estimated share is 18.1% in 2024	**2025**	18%	**2020**	10%	2024	18.10%	101.25%	80.00%	**126.56 %**	**2**	**2**	**1.0328125**	**ACHIEVED**
By 2025, approximately 40 GW of additional hydropower capacity will be installed compared to 2020 (370 GW).	Power sector	The Action Plan for Carbon Peaking Before 2030<br>14th Five-Year Plan for Renewable Energy Development	State Council	By 2024, operational capacity: 436 GW hydropower capacity in total: 377 GW conventional hydro + 58.7 GW pumped storage, under construction capacity: 67 GW, according to the National Bureau of Statistics (NBS).	**2025**	470	**2020**	370	2025	503	133.00%	100.00%	**133.00 %**	**3**	**1**	**1.065**	**ACHIEVED**

By 2025, CO_2 emissions per unit of industrial added value will be reduced by 18%.	Industry	14th Five-Year Plan on Industrial Green Development	Ministries	No evidence for public source, we estimated progress toward the 18% reduction target for CO_2 emissions per unit of industrial value added by combining emissions and economic data. Total CO_2 emissions were drawn from the Global Carbon Project and IEA, with industry assumed to account for 65–70% of energy-related emissions. Industrial value added was taken from NBS and indexed to 2020 = 100. Dividing estimated industrial CO_2 by real value-added yields an intensity index, showing a ~10% decline by 2024 progress, but still short of the 2025 goal.	**2025**	-18%	**2020**	0	2024	-10%	55.56%	80.00%	**69.44%**	**1**	**2**	**0.647222222**	**LOW**

By 2025, energy consumption per unit of industrial added value in large-scale industries will decrease by 13.5% compared to 2020.	Industr y	Action Plan for Industrial Energy Efficiency Improvement<br>The Implementation Plan for Carbon Peaking in the Industrial Sector	Ministries	No evidence, we approximate industrial energy intensity as the ratio of industrial energy use to real industrial value added, indexed to 2020 = 100. Since detailed sectoral data are unavailable, we use NBS data showing industrial value added grew ~29% (2020–2024) and official reports that economy-wide energy intensity fell ~8%. Assuming industrial intensity follows a similar or slightly faster trajectory, we estimate a ~8–11% reduction from 2020 to 2024, suggesting progress but still short of the 13.5% reduction target for 2025.	**2025**	-13.50%	**2020**	0	2024	-10%	74.07%	80.00%	**92.59%**	**1**	**1**	**0.662962963**	**LOW**
By 2025, the proportion of EAF in iron and steel production will increase to 15%.	Industr y	Implementation Plan for Synergizing the Reduction of Pollution and Carbon Emission<br>2024-2025 Energy Conservation and Carbon Reduction Action Plan<br>Guideline on promoting the high-	NDRC	CREA estimates in 2025, While China set a 15% electric arc furnace (EAF) steel share target for 2025, aimed at reducing the emissions of the country's second-largest carbon-	**2025**	15%	**2020**	10%	2024	11%	20.00%	80.00%	**25.00%**	**2**	**1**	**0.425**	**LOW**

		quality development of the iron and steel industry<br>The Implementation Plan for Carbon Peaking in the Industrial Sector		emitting industry, the actual share has remained stagnant at around 10% for over a decade. (Reuters reported China's lag behind in green industry too in 2024)													
By 2025, recycled steel use will reach 320 million tons (updated to 300 million tons in the latest 2024 policy).	Industry	14th Five-Year Plan for Circular Economy Development<br>2024-2025 Energy Conservation and Carbon Reduction Action Plan	NDRC	China recycled 260 MMt of scrap steel in 2023 (210mmt in 2020)<br>GMK Centre reported that China's scrap consumption for 2024 was predicted to be 209.7 million tonnes, which is a 1.9% decrease year-on-year	**2025**	320	**2020**	210	2024	209.7	-0.27%	80.00%	**-0.34%**	**2**	**2**	**0.398295455**	**LOW**
By 2025, domestic primary crude oil refining capacity will be kept below 1 billion metric tons (20 million b/d), and the utilization rate of production capacity for main products will rise to 80% or more.	Industry	The Action Plan for Carbon Peaking Before 2030<br>2024-2025 Energy Conservation and Carbon Reduction Action Plan<br>Special Action Plan for Energy Conservation and Carbon Reduction in the Oil Refining Industry	State Council	In 2023, the country's refining capacity stands at more than 18.29 million b/d, surging by more than 50% from around 12 million b/d in 2022.<br>S&P Global / ETRI report refining capacity of ~961 million mt/year (≈19.3 million b/d) in 2024.<br>Since this is an upper-bound capacity target, we assume capacity remains within the cap, as the	**2025**	10	**2020**	8.9	2024	9.5	100.00%	80.00%	**125.00%**	**3**	**1**	**1.025**	**ACHIEVED**

				first four years did not breach it.														
By 2025, comprehensive energy consumption per ton of steel will be reduced by over 2% compared to 2020 (updated in 2024 to a 2% reduction compared to 2023).	Industry	Guideline on promoting the high-quality development of the iron and steel industry<br>14th Five-Year Plan for the Development of the Raw Materials Industry	Ministries	In 2022, the energy intensity of iron and steel increased by 1.7%	**2025**	-2%	**2020**	0	2022	1.70%	-85.00%	40.00%	**-212.50%**	**1**	**1**	**-0.8625**	**LOW**
By 2025, electricity will account for approximately 30% of industrial end-use energy consumption.	Industry	Action Plan for Industrial Energy Efficiency Improvement	Ministries	From the China Electrification Annual Development Report (September 2025),industrial electrification rate has increased to 27.7%(27% in 2020 from IEA)	**2025**	30%	**2020**	27%	2024	28%	23.33%	80.00%	**29.17%**	**1**	**1**	**0.345833333**	**LOW**

By 2025, the carbon intensity of road transportation will be reduced by 5% compared to 2020.	Transport	2024-2025 Energy Conservation and Carbon Reduction Action Plan<br>The "14th Five-Year Plan for the Development of Modern Comprehensive Transportation System	State Council	No evidence, to track progress toward the target of reducing transportation carbon intensity by 5% between 2020 and 2025, we construct a proxy indicator combining emissions and transport activity. Transport CO_2 emissions, derived from oil product consumption, rose modestly from 1.1 Gt in 2020 to 1.2 Gt in 2024 (IEA, 2025). Over the same period, passenger and freight turnover expanded by an estimated 20–25% as mobility recovered from the pandemic (NBS; Ministry of Transport). Dividing emissions by transport activity shows that carbon intensity in 2024 was 8% lower than in 2020, indicating that the sector has already surpassed the 2025 target ahead of schedule.	**2025**	-5%	**2020**	0	2024	-8%	160.00%	80.00%	**200.00 %**	**3**	**2**	**1.5**	**ACHIEVED**

By 2025, the average electricity consumption of new passenger BEVs will be ≤12.0 kWh/100 km.	Transport	New Energy Automobile Industry Development Plan (2021-2035)	State Council	From the investigation of International Council on Clean Transportation in 2023, the technology efficiency of BEVs in China's real-world ranges from 8.8-14.1 kwh/km. The target level is within the range. Diselnet estimates that in 2022, the average energy consumption of domestic BEVs reached 12.35 kWh/100 km.	**2025**	12 kwh/100	**2020**	-	2025	1.35 Kwh/100km	100.00%	100.00%	**100.00%**	**3**	**1**	**0.9**	**ACHIEVED**
By 2025, the average fuel consumption of new passenger cars will be reduced to 4.0 liters/100 km.	Transport	Mandatory National Standard: Fuel Consumption Limits for Passenger Cars (GB 19578-2021)	Ministry	As it is mandatory standard, we assumed it could be fully implemented	**2025**	4 liters/100 km	**2020**	-	2025	4 liters/100km	100.00%	100.00%	**100.00%**	**1**	**1**	**0.7**	**ACHIEVED**
By 2025, new energy buses will account for 72% of all surface public transport vehicles in urban areas.	Transport	The "14th Five-Year Plan for the Development of Modern Comprehensive Transportation System	State Council	From Ministry of Transport, New energy buses have accounted for 74.1% of all surface public transport vehicles in urban areas as of the end of 2024.	**2025**	72%	**2020**	60%	2024	74.10%	117.50%	80.00%	**146.88%**	**3**	**2**	**1.234375**	**ACHIEVED**

By 2025, sales of new energy vehicles will account for about 20% of total new car sales.	Transport	14th Five-Year Plan on Modern Energy System Planning<br>The "14th Five-Year Plan" Comprehensive Work Plan for Energy Conservation and Emission Reduction<br>New Energy Automobile Industry Development Plan (2021-2035)	NDRC	According to IEA data, the sales share is 38% in 2023(11% in 2020)	**2025**	20%	**2020**	11%	2023	38%	300.00%	60.00%	**500.00%**	**2**	**2**	**2.9**	**ACHIEVED**
By 2025, electricity consumption will account for over 55% of building energy consumption.	Building	14th Five-Year Plan for Building Energy Efficiency and Green Building Development	Ministries	From the China Electrification Annual Development Report， Building sector electrification rate has increased to 55.3% in 2024(44.1% in 2020)	**2025**	55%	**2020**	44%	2024	55.30%	102.75%	80.00%	**128.44%**	**1**	**2**	**0.942201835**	**ACHIEVED**
By 2025, renewable energy use in urban buildings will reach 8%.	Building	The Action Plan for Carbon Peaking Before 2030<br>2024-2025 Energy Conservation and Carbon Reduction Action Plan<br>14th Five-Year Plan for Building Energy Efficiency and Green Building Development<br>Implementation plan for carbon peaking in urban and rural construction	State Council	No evidence, assume China is approaching this target and by 2023, the progress is 50%	**2025**	8%	**2020**	4%	2024	-	50.00%	80.00%	**62.50%**	**3**	**2**	**0.8125**	**MEDIUM**

By 2030, CO_2 emissions per unit of GDP will be reduced by over 65% from the 2005 level.	Overall	China's Achievements, New Goals and New Measures for Nationally Determined Contributions<br>Working Guidance for Carbon Dioxide Peaking and Carbon Neutrality in Full and Faithful Implementation of the New Development Philosophy<br>The Action Plan for Carbon Peaking Before 2030<br>14th Five-Year Plan on Modern Energy System Planning	CCP & State Council	According to IEA data, China's carbon intensity has been lowered by 50% from the 2005 level<br>UNFCCC reports that China's carbon intensity (CO_2/GDP) was down 48.4% vs 2005 by 2020.<br>Reuters reports that China cut carbon intensity a further ~8% cumulatively from 2020 to 2024.After calculation, by 2024, China's carbon intensity was about 52.5%	**2030**	-65%	**2005**	0	2024	-53%	80.77%	76.00%	**106.28 %**	**3**	**2**	**1.031376518**	**HIGH**
By 2030, the share of non-fossil fuels in primary energy consumption will reach around 25%.	Overall	China's Achievements, New Goals and New Measures for Nationally Determined Contributions<br>Working Guidance for Carbon Dioxide Peaking and Carbon Neutrality in Full and Faithful Implementation of the New Development Philosophy<br>The Action Plan for Carbon Peaking Before 2030<br>14th Five-Year Plan on Modern Energy System Planning	CCP & State Council	By 2023, the share of non-fossil fuels in primary energy consumption has increased to 17.5%, according to mid-term evaluation report of the National Development and Reform Commission (15.3% in 2020)<br>By 2024, the proportion of non-fossil energy in primary energy consumption reached 19.7%	**2030**	25%	**2020**	15.90%	2024	19.70%	41.76%	40.00%	**104.40 %**	**3**	**2**	**1.021978022**	**HIGH**

By 2030, the forest coverage rate will reach about 25%, and the forest stock volume will reach 19 billion cubic meters.	Overall	Working Guidance for Carbon Dioxide Peaking and Carbon Neutrality in Full and Faithful Implementation of the New Development Philosophy<br>The Action Plan for Carbon Peaking Before 2030	CCP & State Council	China daily reported that the forest coverage rate surpassed 25 percent, while the forest stock volume exceeded 20 billion cubic meters, by 2025	**2030**	**19**	**2020**	17.56	2024	20	169.44%	40.00%	**423.61 %**	**3**	**2**	**2.618055556**	**ACHIEVED**
By 2030, approximately 40 GW of additional hydropower capacity will be installed compared to 2025 (370 GW).	Power sector	The Action Plan for Carbon Peaking Before 2030<br>14th Five-Year Plan of Renewable Energy	State Council	Installed hydro capacity had climbed 18% to 422 million kilowatts (kW) by the end of 2023 from 358 million kW at the end of 2019, according to the National Bureau of Statistics (NBS).<br>By 2024, operational capacity: 436 GW hydropower capacity in total: ~377 GW conventional hydro + ~58.7 GW pumped storage, under construction capacity: ~67 GW of additional hydro projects (≈40 GW conventional + ≈27 GW pumped storage.	**2030**	510	**2020**	370	2025	503	95.00%	50.00%	**190.00 %**	**3**	**2**	**1.45**	**HIGH**

By 2030, the total installed capacity of wind and solar power will exceed 1,200 GW.	Power sector	China's Achievements, New Goals and New Measures for Nationally Determined Contributions<br>Working Guidance for Carbon Dioxide Peaking and Carbon Neutrality in Full and Faithful Implementation of the New Development Philosophy<br>The Action Plan for Carbon Peaking Before 2030<br>14th Five-Year Plan of Renewable Energy	CCP & State Council	Guardian shows, China is on track to reach **1,200GW** of installed wind and solar capacity by the end of 2024, six years ahead of the government's target.	**2030**	1200	**2020**	535	2025	1673	171.13%	50.00%	**342.26 %**	**3**	**2**	**2.211278195**	**ACHIEVED**
By 2030, around 40% of incremental vehicles will be fuelled by new and clean energy.	Transp ort	The Action Plan for Carbon Peaking Before 2030<br>Implementation Plan for Synergizing the Reduction of Pollution and Carbon Emission	State Council	According to IEA data, the sales share is 38% in 2023(11% in 2020)	**2030**	40%	**2020**	11%	2023	38%	93.10%	30.00%	**310.34 %**	**3**	**1**	**1.951724138**	**HIGH**
By 2030, petroleum consumption for land transportation will peak before 2030.	Transp ort	The Action Plan for Carbon Peaking Before 2030	State Council	While direct evidence is limited, given the strong progress on other transport sector targets, we assume this goal is likely to be achieved, with a peak expected around 2030.<br>The IEA notes that China's consumption of combustion fuels (gasoline, diesel, jet fuel) declined slightly	**2030**	peak	**2020**	-	2030	peak	99.00%	100.00%	**99.00%**	**3**	**2**	**0.995**	**HIGH**

				in 2024, despite a rebound in aviation. This suggests road/transport fuel use may have already plateaued.												
By 2030, the average fuel consumption of new passenger cars will be reduced to 3.2 liters/100 km.	Transport	Mandatory National Standard: Fuel Consumption Limits for Passenger Cars (GB 19578-2021)	Ministries	As it is mandatory standard, we assumed it could be fully implemented	**2030**	3.2 litres/100 km	**2020**	-	2030	-	100.00%	100.00%	**100.00 %**	**1**	**1**	**0.7**	**ACHIEVED**
By 2030, 100% of public vehicles will be electrified.	Transport	14th Five-Year Plan of Green Transportation Development<br>Action Plan for Continuous Improvement of Air Quality<br>New Energy Automobile Industry Development Plan (2021-2035)	State Council	From Ministry of Transport, New energy buses have accounted for 74.1% of all surface public transport vehicles in urban areas<br>as of the end of 2024.	**2030**	100%	**2020**	60%	2024	74.10%	35.25%	40.00%	**88.13%**	**3**	**2**	**0.940625**	**HIGH**
By 2030, the proportion of EAF in iron and steel production will increase to over 20%.	Industry	Implementation Plan for Synergizing the Reduction of Pollution and Carbon Emission<br>The Implementation Plan for Carbon Peaking in the Industrial Sector	Ministries	CREA estimates in 2025, While China set a 15% electric arc furnace (EAF) steel share target for 2025, aimed at<br>reducing the emissions of the country's second-largest carbon-emitting industry, the actual share has	**2030**	20%	**2020**	10%	2024	11%	10.00%	40.00%	**25.00%**	**1**	**1**	**0.325**	**LOW**

				remained stagnant at around 10% for over a decade. (Reuters reported China's lag behind in green industry too in 2024)													
By 2030, electricity will account for over 65% of building energy consumption.	Buildin g	Implementation plan for carbon peaking in urban and rural construction	Ministries	From the China Electrification Annual Development Report， building sector electrification rate has increased to 55.3% in 2024(((44.1% in 2020)	**2030**	65%	**2020**	44%	2024	55.30%	53.59%	40.00%	**133.97 %**	**1**	**1**	**0.869856459**	**MEDIUM**
By 2035, the share of non-fossil fuels in total energy consumption will exceed 30%.	Overall	NDC 2035	CCP & State Council	By 2023, the share of non-fossil fuels in primary energy consumption has increased to 17.5%, according to mid-term evaluation report of the National Development and Reform Commission (15.3% in 2020)<br>By 2024, the proportion of non-fossil energy in primary energy consumption reached 19.7%	**2035**	30%	**2020**	15.30%	2024	19.70%	29.93%	26.67%	**112.24 %**	**3**	**2**	**1.06122449**	**HIGH**
By 2035, the total installed capacity of wind and solar power	Power sector	NDC 2035	CCP & State Council	Guardian shows, China is on track to reach **1,200GW** of installed wind and solar capacity by the	**2035**	3600	**2020**	535	2024	1407	28.45%	26.67%	**106.69 %**	**3**	**2**	**1.033442088**	**HIGH**

generation will reach 3,600 GW, more than six times the 2020 level.				end of 2024, six years ahead of the government's target.													
By 2060, the share of non-fossil fuels in energy consumption will exceed 80%.	Overall	China's Mid-Century Long-Term Low Greenhouse Gas Emission Development Strategy<br>Working Guidance for Carbon Dioxide Peaking and Carbon Neutrality in Full and Faithful Implementation of the New Development Philosophy	CCP & State Council	By 2023, the share of non-fossil fuels in primary energy consumption has increased to 17.5%, according to mid-term evaluation report of the National Development and Reform Commission (15.3% in 2020)	**2060**	80%	**2020**	15.30%	2023	17.50%	3.40%	7.50%	**45.34%**	**3**	**1**	**0.626687275**	**LOW**

SI Section 2: Morphological analysis of China's energy transformation

SI Table 4.Morphological table of scenario development

**Economy development**	**Low Growth: Prolonged Stagnation Due to Internal and External Pressures**<br>China's economic growth is constrained by both external and internal challenges. Declining global demand, rising tariffs, high debt levels, restricted investment, and weak domestic consumption collectively hinder economic expansion. Macroeconomic policies fail to effectively mitigate these pressures, resulting in prolonged economic stagnation.<br><br>**GW**	**Moderate Growth: Industrial Support Amid Structural Challenges**<br>China's role as the "world factory" and the ongoing urbanization continue to support the development of traditional energy-intensive industries, contributing to economic growth. However, the investment-driven growth model is becoming increasingly unsustainable. High debt levels and an overly competitive labour market erode consumer confidence and suppress domestic spending, limiting future growth potential.<br>**CS**	**High Growth Driven by Real Estate Recovery and New Urbanisation**<br>To mitigate the crisis in the real estate market, the government lifts previous restrictions on the property sector, encouraging developers to complete ongoing projects. Real estate recovery and new urbanisation make energy and resource-intensive industries, remain a critical pillar of the economy, driving robust economic growth.<br><br>RS	**High Growth Driven by Green and High-Tech Manufacturing**<br>Economic reforms have strengthened the domestic market by increasing the share of household income in GDP, thereby stimulating consumer spending. Expanding domestic and international consumption drives the growth of green manufacturing, high-tech industries, and the service sector, which collectively replace real estate as the new pillars of sustainable economic development.<br>**GL**
**Demographic changes**	**Alleviated decline: Social reforms and Economic Prosperity Drive Family Formation**<br>The complete abolition of population control policies, coupled with the implementation of hukou system reforms, could significantly enhance	**Moderate decline: Rising Costs and Social Shifts Suppress Fertility**<br>With continued economic growth and rising per capita income, the costs of housing, education, healthcare, and childcare have significantly increased, making child-rearing more expensive.	**Accelerated decline: Economic Instability Undermines fertility intension**<br>Economic downturns lead to a deteriorating employment environment, undermining the confidence of younger generations. Additionally, concerns about healthcare, elderly care, education, and	

	societal willingness to have children. Economic prosperity further boosts the confidence of younger generations in family formation.<br><br>**RS**	Furthermore, greater gender equality and women's empowerment have led to increased workforce participation, reducing societal pressure to have larger families.<br>**CS GL**	food safety make young people more cautious in decisions related to marriage and childbearing.<br><br>**GW**	
**National Climate Policy**	**Limited Progress Beyond Current Achievements**<br>China struggles to advance beyond its existing decarbonization accomplishments. While significant progress has been made in renewable energy and electric vehicle deployment, economic downturns and technological constraints in industrial decarbonization hinder further policy implementation. This results in significant uncertainty regarding the achievement of both NDC targets and the 2060 net-zero goal.<br>**GW**	**NDC Achievable, Net-Zero by 2060 Uncertain**<br>Building on progress in renewables and electric vehicles, rising household consumption drives electrification across end use sectors, enabling the effective achievement of electricity-related policy goals. However, the economy's reliance on manufacturing and energy-intensive industries weakens the implementation of industrial emission reduction policies. While achieving the NDC target is feasible, meeting the net-zero target by 2060 remains highly uncertain.<br>**RS**	**Policy Continuity Secures Climate Targets**<br><br>All existing climate policies are fully implemented, and sectoral goals are consistently met. China effectively enforces climate policies across sectors through the 2030s and maintains sustained decarbonization efforts beyond 2030. As a result, China successfully reaches its carbon peak by 2030 and achieves carbon neutrality by 2060 as planned.<br><br>**CS**	**Ambitious Policies Drive Early Success**<br><br>China emerges as a global leader in climate change mitigation, achieving its NDC and net-zero targets ahead of schedule through the implementation of more ambitious and comprehensive policies.<br><br>**GL**

**Clean technology deployment**	**Limited Innovation and Advancement: Slow Economic Growth Hinders Technological Progress**<br>Economic slowdown limits investment in research and development, resulting in slow innovation and technological advancement. Cost reductions in low-carbon technologies, such as EVs, wind and solar power, progress slowly.<br>**GW**	**Moderate Advancements: Gradual but Steady Technological Progress**<br>Incremental advancements in low-carbon technologies are driven by economic growth or supported by government climate priorities. While the costs of renewable energy technologies, such as wind, solar, and energy storage, continue to decline steadily. the development and deployment of CCS, BECCS, and DAC technologies face persistent scalability challenges, pushing full commercialization beyond 2040.<br>**CS**	**Breakthrough Innovation: Economic Growth Accelerates Technological Leadership**<br>Strong economic growth drives significant investment in the research, development, and deployment of low-carbon technologies. Costs of renewable technologies—including wind, solar, and energy storage—decline rapidly.<br>**RS, GL**	
**CCS deployment**	**Low-End Official Estimates Reflect Limited Government Climate Ambition**<br>The deployment of Carbon Capture and Storage (CCS), including Bioenergy with CCS (BECCS) and Direct Air Capture (DAC), is significantly delayed in this scenario. Limited policy support and lower climate ambition hinder large-scale investment and adoption, resulting in	**Medium Official Estimates Reflect Mixed Drivers of Technological Development**<br>The development and deployment of CCS, BECCS, and DAC technologies advance gradually due to a combination of economic stimulus or government-led innovation efforts. However, persistent challenges in cost, infrastructure, and scalability	**High-End Official Estimates Reflect Strong Government Ambition:**<br>CCS achieves full commercialisation by the 2030s, BECCS scales up significantly by 2040, and DAC sees widespread deployment by 2050—enabling large-scale carbon removal and contributing substantially to national emissions reductions.	

	slow technology development and deployment through mid-century.<br>**GW**	delay widespread commercialisation until after 2040.<br><br>**RS, CS**	**GL**	

SI Section 3 : China energy transformation scenarios

Green Lights Scenario

Green Lights(GL) portrays a future of high economic growth and green development in China, mean “go, advance and carry on”[20]. China emerges as a global model for sustainable development and climate leadership by 2060. Through a strategic blend of economic reform, technological innovation, ambitious climate policies, and active public participation, China successfully meets and surpasses its climate targets. The Green Lights scenario demonstrates that climate actions could be well taken into an economic opportunity[21], highlighting China’s path to a greener, more resilient future where innovation, policy, and societal commitment converge.

In the Green Lights scenario, China embarks on a transformative path toward sustainable and inclusive economic growth following its **Dual Circulation Development Paradigm**[22] and **Beautiful China Initiative (BCI)**[23]. Central to dual circulation, China will “take the domestic circulation as the mainstay”, expend domestic expending and reduce reliance on investment-led growth[24]. This rebalanced economic model fosters industrial diversification, with green and high-tech manufacturing and service sector becoming the core drivers of growth.

As one of the main requirements for achieving a Beautiful China, China is ‘actively and steadily promoting carbon peak and carbon neutrality’[25]. Building on its **1+N Climate Policy System**, the government enacts even more ambitious and comprehensive climate policies that go beyond its **Nationally Determined Contributions (NDCs)** and enable the country to achieve net-zero emissions by 2050—ten years ahead of schedule.

This policy’s success is underpinned by rapid advancements in low-carbon technologies. Driven by **Made in China 2025**[26], the costs of clean energy technologies—including electric vehicles, wind turbines, solar PVs—decline rapidly[27]. The strategic target of cultivating **New Quality**

Productive Forces (NQPFs) will drive large-scale investments in research, development, and deployment of innovative technologies[28]. Technological breakthroughs and commercial operations of key carbon removal technologies are further supported by government programs such as “973 program” and “863 program”[29] . Significant advancements in carbon removal technologies are achieved: China's CCS emissions reduction (including BECCS and DAC) reaches 408 million tons by 2030, 1.45 billion tons by 2050, and 1.82 billion tons by 2060[30]. Direct Air Carbon Capture is expected to begin industrial demonstration and promotion by 2035.

Strong government policies and technological breakthroughs drive significant shifts in public environmental behaviour. Sustainable lifestyles become mainstream, driven by demand-side energy reductions, community-led environmental actions, and the adoption of decentralized low-carbon technologies. Programs such as trade-in schemes[31] and local government consumption vouchers[32] further accelerate the widespread adoption of green home appliances, enhancing energy efficiency and reducing household emissions.

As the share of household spending in GDP increases, it reflects rising incomes and improved living standards, which give young people greater confidence in their future. However, the accompanying rise in living costs—encompassing housing, education, healthcare, and childcare—discourages family formation. Consequently, fertility rates continue to decline moderately, following current trends.

Red Sun Scenario

In traditional Chinese culture, the colour red symbolizes celebration and prosperity. The image of a rising red sun evokes a sense of improving living standards and growing happiness among the people. The Red Sun (RS) scenario envisions a vibrant and thriving Chinese economy, with a primary focus on economic development, while achieving climate commitments is not the government's top priority. By 2060, China remains a global economic leader, but its environmental progress is fragmented. While the country successfully meets its NDC goals by 2030, the reliance on heavy industries and an investment-driven society hampers progress toward the 2060 net-zero goal. Technological advancements contribute modestly to emissions reductions, but the lack of transformative policy measures and limited public engagement in sustainability prevent comprehensive climate action. The Red Sun scenario underscores the risks of prioritizing short-term economic growth over long-term environmental sustainability, leaving China vulnerable to future environmental challenges and economic instability.

In the Red Sun scenario, China prioritizes economic growth by **real estate market recovery**[33] and **people-centred new urbanization strategy**[34]

To reverse the downturn of the property market, the government lifts previous restrictions and rolls out a bundle of pro-housing policies since 2024, unveils new measures to stabilize housing market encouraging developers to resume and complete stalled projects[35]. Urban

agglomerations is consistently emphasized as key drivers of economic growth from China's 11th Five-year Plans[36,37]. As a developing country, China's urbanization rate reached 66.16% by the end of 2023, leaving considerable room for growth compared to developed nations such as the United States and the United Kingdom, where urbanization levels are around 85%[38]. The people-centred new urbanization strategy proposed in the **14th Five-Year Plan** further emphasizes "promoting the urbanization of the agricultural transfer population as the primary task of new urbanization"[39],which will stimulate substantial investment in municipal and rural infrastructure, public services, and housing, creating substantial new investment opportunities with the potential to drive GDP growth.

Although the new urbanization strategy also mentioned "strengthening ecological restoration and environmental protection" [39], this growth-first approach drives increased industrial production and infrastructure expansion, leaving the government with limited resources to allocate toward industrial decarbonization and technological innovation. The costs of clean energy technologies, such as electric vehicles, wind, and solar, continue to decline steadily, enabling China to meet its NDC target by 2030. However, the development and deployment of carbon removal technologies—Carbon Capture and Storage (CCS), Bioenergy with Carbon Capture and Storage (BECCS), and Direct Air Capture (DAC)—face persistent scalability and commercialization challenges. Without more aggressive policy incentives, achieving the net-zero target by 2060 remains highly uncertain.

Demographic trends in the Red Sun scenario are influenced by a series of government-led social reforms. The continuous decline in fertility rates has been alleviated since the implementation of the three-child policy in 2021[40]. Reforms to the *hukou* (household registration) system, including improved guarantees for migrant children and relaxed settlement restrictions in cities[41], significantly reduce childbearing and education costs, enhancing societal fertility intension. Additionally, economic prosperity boosts younger generations' confidence in family formation, further delaying population shrinking.

Consumer behaviour in this scenario is shaped by rising incomes and an increasing preference for comfort and convenience. Higher disposable income drives increased spending on commercial and entertainment activities, resulting in continuous growth in energy service demand (e.g., tourism)[42]. While green technologies such as electric vehicles and energy-efficient appliances see widespread adoption, this shift is largely motivated by affordability and government incentives (e.g., trade-in schemes[31] and local government consumption vouchers[32]) rather than genuine environmental consciousness. Environmental awareness remains low, with sustainability taking a backseat to personal comfort and consumption.

Calm Sea Scenario

The Calm Sea (CS) scenario offers a middle-of-road benchmark for the future, both in terms of socio-economic development and climate ambitions, like a 'sea level.' It explores the potential transition of the Chinese energy system, assuming full implementation of current climate policies and successful achievement of climate targets, amidst moderate economic and population changes. By 2060, China achieves its core climate objectives, including peaking emissions by 2030 and reaching carbon neutrality by 2060, in alignment with its international commitments. However, the trade-off between economic growth and emissions reduction results in progress being made through steady, incremental advancements rather than transformative change.

In Calm Sea Scenario, China is pursuing a path toward long-term sustainable economic development, aiming to balance economic growth with its climate commitments. The emerging "new three" industries[43]—solar power, electric vehicles (EVs), and batteries—have become not only key drivers of China's economy but also significant contributors to its decarbonization objectives[44]. However, the rapid expansion of clean energy investments introduces the risk of overcapacity, exacerbating structural challenges inherent in China's investment-driven economic model. To address weak domestic and international demand and the lingering effects of the real estate bubble, the Chinese government has implemented a Dual Circulation Strategy aimed at stimulating domestic consumption and fostering international cooperation. This approach seeks to create new avenues for investment demand, mitigate economic slowdowns, and support sustained moderate growth.

A relatively optimistic economic outlook provides the government with greater flexibility to uphold its climate commitments. However, policy measures lack the boldness necessary to drive more accelerated transitions. China demonstrates policy stability by fully implementing existing climate policies and consistently meeting sectoral targets, enabling the country to peak carbon emissions by 2030 and remain on track to achieve carbon neutrality by 2060.

Achieving dual carbon targets requires additional technological support, encompassing both low-carbon technologies and carbon removal solutions. The costs of clean energy technologies such as electric vehicles (EVs), wind, solar, and energy storage continue to decline steadily, accelerating the adoption of green technologies in household. Public behaviours in this scenario reflects passive adaptation to policy-driven changes rather than proactive engagement in sustainability[45]. According to estimates from the Ministry of Ecology and Environment, CCS emissions reduction (including BECCS and DAC) is expected to reach at least 20 million tons by 2030, 600 million tons by 2050, and 1 billion tons by 2060. DAC is projected to begin industrial demonstration and promotion by 2040[30].

Great Wall Scenario

Historically, the Great Wall symbolized China's military defence during times of danger. In the Great Wall (GW) scenario, it represents the barriers and challenges that hinder significant and rapid transformations in China's economy and energy system. In this context, China faces compounded economic, demographic, and environmental challenges. The stagnating economy, declining population, and stalled climate progress leave the country struggling to sustain growth and meet its international climate commitments. The Great Wall presents a conservative, constrained future where economic hardship and limited policy execution hinder China's ability to transition to a sustainable and resilient economy.

In the Great Wall scenario, China faces prolonged economic stagnation due to property market crisis[46] and sluggish consumer spending[47]. After decades of investing in infrastructure and real estate at breakneck speed, China has likely reached the point of sharply diminishing returns[48]. The existing export- and investment-led growth model is losing momentum, while transitioning to a consumption-led growth model proves challenging due to high debt levels among local governments (over 90% of GDP) and households (over 60% of GDP) [49,50].

The economic downturn significantly affects social norms surrounding fertility intentions and sustainable lifestyles. A weakening job market, combined with rising concerns over healthcare, elderly care, education, and food safety, undermines the confidence and trust of younger generations in pursuing marriage and family formation[51]. Pro-natalist policies fail to reverse the declining fertility rate, contributing to long-term labour market strain and reduced consumer spending. The focus for the majority shifts toward achieving a stable and secure standard of living, with limited attention given to energy conservation or sustainability.

Technological innovation slows markedly due to reduced funding for research and development. Cost reductions in low-carbon technologies, such as electric vehicles, wind, and solar power, stagnate. The deployment and commercialization of critical carbon removal technologies—CCS, BECCS, and DAC—are significantly delayed, preventing large-scale implementation.

China's progress in decarbonization stalls, falling "off track on all its core 2025 climate targets"[52,53] and remaining constrained to earlier achievements in renewable energy deployment and electric vehicle adoption. Industrial decarbonization efforts falter due to financial constraints, which limit large-scale investments in clean energy infrastructure and emissions reduction technologies. Overall, economic stagnation and technological limitations hinder further policy advancements, casting significant doubt on the country's ability to meet its NDC target by 2030 and its net-zero goal by 2060.

SI Section 4: Scenario quantification and key assumptions

This supplementary information outlines the core assumptions underpinning our analysis. We develop a set of evidence-based model inputs to operationalize the above scenarios within the TIAM-UCL integrated assessment model. Below, we present the key quantified metrics used to translate the scenario narratives into model inputs:

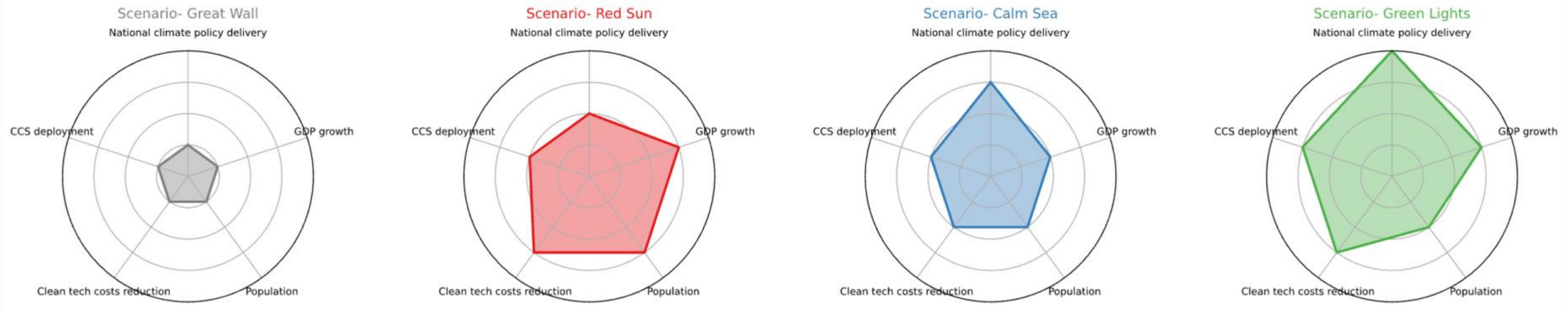

SI Figure 2.key scenario quantification metrics. To quantify the scenario narratives in TIAM-UCL, we examine five key metrics and their associated uncertainties in this section, along with the corresponding modelling assumptions: national climate policy delivery, GDP growth, population, clean technology cost reductions, and CCS deployment rates. The degree of national climate policy delivery from low to high reflects the implementation of policies from ‘achieved’ to ‘low credibility’ levels. Numerical assumptions for the corresponding quantification metrics are detailed in following sections.

4.1 National Climate policy delivery

Based on national climate action narratives, we use the degree of policy delivery as a quantitative indicator across scenarios (see SI Figure 2). This is determined by the assessed credibility level of each target—classified as ‘achieved’, ‘high’, ‘medium’, or ‘low’. Targets with higher credibility ratings are more likely to be implemented in practice. Accordingly, the Great Wall scenario, which enforces only ‘achieved’ targets, represents the most conservative pathway, reflecting minimal delivery. Across scenarios, targets are progressively implemented from high to low credibility. From Great Wall to Green Lights, the strength of policy implementation increases (SI Table 5), with an expanding set of targets

assumed to be met (SI Table 6), culminating in the full realisation of China’s overarching climate commitment: carbon neutrality by 2060 or before 2060 (assumed as 2050).

For targets that are already achieved or even overachieved, we implement the actual realised levels by 2025 rather than the stated target levels to better reflect China’s existing climate efforts. For example, this applies to indicators such as EV market share or installed solar and wind capacity. After the target year, we assume that these achieved levels will not decline; instead, they are maintained as the minimum baseline from 2025 onwards. As additional targets are implemented from Great Wall to Green Lights, some previously achieved targets are further raised to align with higher-level, more normative goals (e.g., non-fossil share, net-zero commitment). A clear example can be seen in the Calm Sea and Green Lights scenarios: once the net-zero target is introduced, the “achieved” policy levers define the lower bound of the energy system structure (e.g., sectoral electrification level), while the net-zero and other more system-wide targets further strengthen these levers to ensure that the overall pathway is consistent with achieving net zero. This also helps identify the policy gap between the policies currently issued and the commitments made under the net-zero pledge.

SI Table 5.Quantification of national climate actions in TIAM-UCL

**Scenario**	**Great Wall**	**Red Sun**	**Calm Sea**	**Green Lights**
**Narratives around national climate actions**	**Limited Progress Beyond Current Achievements**<br>China struggles to advance beyond its existing decarbonization accomplishments. While significant progress has been made in renewable energy and electric vehicle deployment, economic downturns and technological constraints in industrial	**NDC Achievable, Net-Zero by 2060 Uncertain**<br>Building on progress in renewables and electric vehicles, rising household consumption drives electrification across end use sectors, enabling the effective achievement of electricity-related policy goals. However, the economy’s reliance on	**Policy Continuity Secures Climate Targets**<br><br>All existing climate policies are fully implemented, and sectoral goals are consistently met. China effectively enforces climate policies across sectors through the 2030s and maintains sustained decarbonization efforts beyond	**Ambitious Policies Drive Early Success**<br><br>China emerges as a global leader in climate change mitigation, achieving its NDC and net-zero targets ahead of schedule through the implementation of more ambitious and comprehensive policies.

	decarbonization hinder further policy implementation. This results in significant uncertainty regarding the achievement of both NDC targets and the 2060 net-zero goal. **GW**	manufacturing and energy-intensive industries weakens the implementation of industrial emission reduction policies. While achieving the NDC target is feasible, meeting the net-zero target by 2060 remains highly uncertain. **RS**	2030. As a result, China successfully reaches its carbon peak by 2030 and achieves carbon neutrality by 2060 as planned. **CS**	**GL**
**Degree of Climate policy delivery**	**Achieved targets**	**Targets ranging from those already achieved to those with high credibility**	**Targets ranging from those already achieved to those with medium credibility**	**Targets ranging from those already achieved to those with low credibility**

SI Table 6.Implementation of policy levers across China's scenarios

Credibility rate	Policy levers	Sceanrio implementation			
		GW	RS	CS	GL
Achived	By 2025, the forest coverage rate will reach 24.1%, and the forest stock volume will rise to 18 billion cubic meters.				
Achived	By 2025, annual crude oil production will stabilize at around 200 million tons.				
Achived	By 2025, annual natural gas production will exceed 230 billion cubic meters.				
Achived	By 2025, 100,000–200,000 tons of hydrogen will be produced annually from renewable energy.				
Achived	By 2025, total installed power generation capacity will reach approximately 3,000 GW.				
Achived	By 2025, the operational installed capacity of nuclear power will reach approximately 70 GW.				
Achived	By 2025, power generation from renewable energy will reach approximately 3,300 TWh.				
Achived	By 2025, 33% of electricity will be generated from renewables.				
Achived	By 2025, 18% of electricity will come from non-hydropower renewables.				
Achived	By 2025, approximately 40 GW of additional hydropower capacity will be installed compared to 2020.				
Achived	By 2025, domestic primary crude oil refining capacity will be kept below 1 billion metric tons (20 million b/d), and the utilization rate of production capacity for main products will rise to 80% or more.				
Achived	By 2025, the carbon intensity of road transportation will be reduced by 5% compared to 2020.				
Achived	By 2025, the average electricity consumption of new passenger BEVs will be $\leq$12.0 kWh/100 km.				
Achived	By 2025, the average fuel consumption of new passenger cars will be reduced to 4.0 liters/100 km.				
Achived	By 2025, new energy buses will account for 72% of all surface public transport vehicles in urban areas.				
Achived	By 2025, sales of new energy vehicles will account for about 20% of total new car sales.				
Achived	By 2025, electricity consumption will account for over 55% of building energy consumption.				
Achived	By 2030, the forest coverage rate will reach about 25%, and the forest stock volume will reach 19 billion cubic				
Achived	By 2030, the total installed capacity of wind and solar power will exceed 1,200 GW.				
Achived	By 2030, the average fuel consumption of new passenger cars will be reduced to 3.2 liters/100 km.				
High	By 2025, the share of non-fossil energy consumption will reach around 20%.				
High	By 2030, CO_2 emissions per unit of GDP will be reduced by over 65% from the 2005 level.				
High	By 2030, the share of non-fossil fuels in primary energy consumption will reach around 25%.				
High	By 2030, approximately 40 GW of additional hydropower capacity will be installed compared to 2025 (370 GW).				
High	By 2030, around 40% of incremental vehicles will be fueled by new and clean energy.				
High	By 2030, petroleum consumption for land transportation will peak before 2030.				
High	By 2030, 100% of public vehicles will be electrified.				
High	By 2035, the share of non-fossil fuels in total energy consumption will exceed 30%.				
High	By 2035, the total installed capacity of wind and solar power generation will reach 3,600 GW, more than six times the 2020 level.				
Medium	By 2025, energy consumption per unit of GDP will be lowered by 13.5% from the 2020 level.				
Medium	By 2025, carbon dioxide (CO_2) emissions per unit of GDP will be lowered by 18% from the 2020 level.				
Medium	By 2025, electricity will account for about 30% of end-use energy consumption.				
Medium	By 2025, total renewable energy consumption will reach about 1 billion tons of standard coal (~29,300 PJ).				
Medium	By 2025, renewable energy will account for more than 50% of the incremental increase in primary energy				
Medium	By 2025, the non-electric utilization of renewable energy (including geothermal heating, biomass heating, biomass fuels, and solar thermal utilization) will reach over 60 million tons of standard coal (~1,758 PJ).				
Medium	By 2025, the proportion of non-fossil energy in power generation will reach about 39%.				
Medium	By 2025, renewable energy use in urban buildings will reach 8%.				
Medium	By 2030, electricity will account for over 65% of building energy consumption.				
Low	By 2025, renewable energy will account for about 18% of primary energy consumption.				
Low	By 2025, CO_2 emissions per unit of industrial added value will be reduced by 18%.				
Low	By 2025, energy consumption per unit of industrial added value in large-scale industries will decrease by 13.5% compared to 2020.				
Low	By 2025, the proportion of EAF in iron and steel production will increase to 15%.				
Low	By 2025, recycled steel use will reach 320 million tons (updated to 300 million tons in the latest 2024 policy).				
Low	By 2025, comprehensive energy consumption per ton of steel will be reduced by over 2% compared to 2020 (updated in 2024 to a 2% reduction compared to 2023).				
Low	By 2025, electricity will account for approximately 30% of industrial end-use energy consumption.				
Low	By 2030, the proportion of EAF in iron and steel production will increase to over 20%.				
Low	By 2060, the share of non-fossil fuels in energy consumption will exceed 80%.				

4.2 Economic growth

China's economic development has exhibited different characteristics across stages, shaped by structural shifts from a labour-intensive economy to one increasingly driven by industry and services, each with varying impacts on energy demand[54,55]. Since the beginning of economic reforms in 1978, China experienced rapid growth, averaging nearly 10% annually between 2000 to 2013 [56]. In the years following, China entered a "new normal,"

transitioning from a growth model focused on speed and scale to one prioritising quality and efficiency [57]. From 2013 to 2019, GDP growth moderated to around 6–7%, constrained by factors such as a shrinking labour force, diminishing returns on investment, and slowing productivity gains. In 2021, the economy was significantly affected by COVID-19 lockdowns and the "zero-COVID" policy, resulting in a sharp downturn. Following the relaxation of pandemic restrictions in 2022, economic activity began to rebound. In 2024, China's GDP grew by 5%, narrowly meeting the government's official target.

Although there are signs of recovery in China's economy following the pandemic, since 2023, challenges such as the real estate slump and decreased demand for exports have impeded its growth to pre-pandemic rates. Major international banks, including Goldman Sachs, UBS, JPMorgan, Morgan Stanley, and CITI, projected China's annual GDP growth rate of 2024 to be between 4.2% and 4.9% [58]. The International Monetary Fund (IMF) forecasts similar growth rates of 4.6% for 2024 and 4.1% for 2025 [49]. By 2028, the growth is expected to decelerate to approximately 3.5%, influenced by challenges such as declining productivity, an aging population, diminishing investment returns, and geoeconomic fragmentation [59]. The Japan Centre for Economic Research (JCER) predicts that China's growth rate will fall below 3% post-2029, potentially declining further if the real estate bubble bursts [60]. Economy scenarios study from Carnegie [61] which analysed China's debt burden, economy driven model (consumption, investment), shows that if China rebalances its economy structure from investment to consumption, the long term (15-20 years) GDP growth rate is limited to 2-3 % due to disruption caused by structural transition.

In addition to commercial projections, many international research institutions produce global GDP forecasts using macroeconomic models that align with various future development narratives. The most widely used framework is the Shared Socioeconomic Pathways (SSPs)[62], which outlines five global scenarios along axes of adaptation and mitigation. These narratives are quantified by research teams to provide projections for GDP, population, and urbanisation. For GDP, three interpretations have been developed by OECD, IIASA, and PIK[63]. Though all follow the SSP storylines, they differ in methodology and assumptions, leading to varying outcomes. However, the SSP framework tends to generalise global development, assuming different degree convergence of income by 2100, often overlooking the country level uncertainties. For example, IIASA's GDP projections show a stagnation trend for China from 2020 to 2040, while OECD's ENV-Growth model suggests ~5% annual growth from 2025 to 2030, then a gradual decline.

Given this divergence and the need for a robust understanding of uncertainties in China's economic development, our study derives three GDP growth trajectories—high, medium,

and low—based on a wide range of sources. These include 267 China national scenarios from IPCC AR6, updated SSP1–5 projections from IIASA and OECD, short-term forecasts from the IMF and major investment banks, as well as scenario inputs from BP, IEEJ, Equinor, Shell, and the IEA (see SI Figure 3). We select the 25th, 50th (median), and 75th percentiles of the GDP projection distribution to represent the low, medium, and high growth trajectories, respectively. These correspond to the China's GDP pathways of the Great Wall (Low), Calm Sea (Medium), Red Sun (High), and Green Lights (High) scenarios.

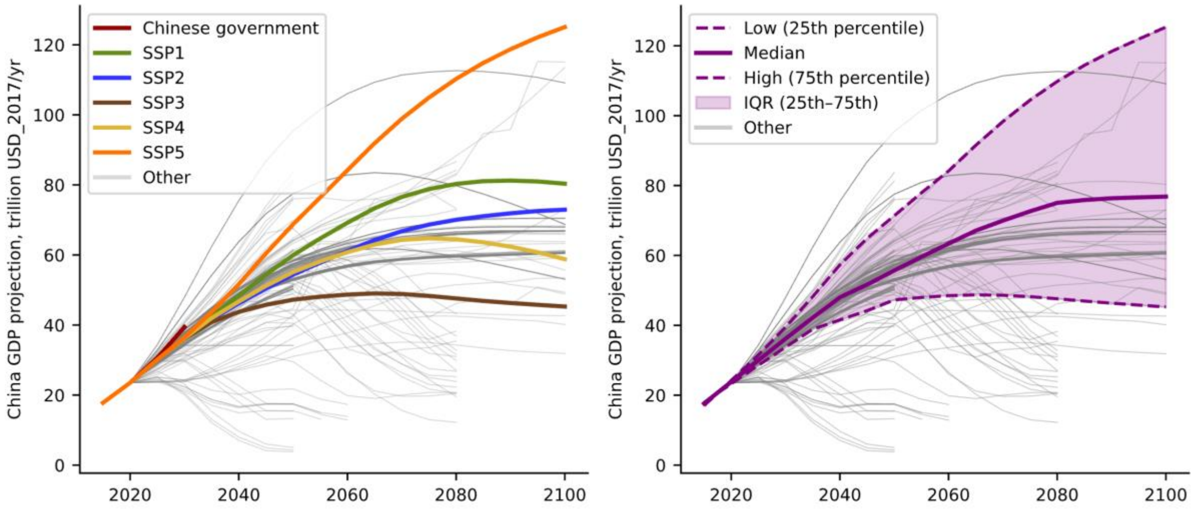

SI Figure 3.China GDP projections uncertainty, other includes: China national scenarios from IPCC AR6, short-term forecasts from the IMF and major investment banks, as well as scenario inputs from BP, IEEJ, Equinor, Shell, and the IEA. (n=267 samples, the low, median, and high trajectories shown have been smoothed to reflect consistent long-term trends)

4.3 Population changes

China has had the world's largest population since at least 1950. Most projections have predicted that its population would peak between 2030 and 2035 at around 1.4–1.5 billion before entering a gradual decline. However, data from China's National Bureau of Statistics revealed that in 2022[64], the population declined to 1.41 billion, marking the first year-on-year drop in six decades. This unexpected shift has prompted widespread debate among scholars and policymakers over the accuracy of earlier projections and the broader implications for China's economic growth, labor force, and social welfare systems.

For future population projections, numerous studies have examined China's demographic outlook, with most drawing on datasets from key institutions such as the World Bank, the United Nations (UN), and the International Institute for Applied Systems Analysis (IIASA)(SI Table 7). The World Bank provides national estimates under varying economic scenarios up to 2050, which is insufficient for the long-term horizon required by our TIAM modelling. The UN publishes global forecasts based on demographic modelling techniques, while IIASA

offers long-term projections (2010–2100) as part of the Shared Socioeconomic Pathways (SSPs) framework.

In this study, we adopt the UN's 2022 population projections for three main reasons. First, although SSP projections are widely used, they do not fully capture recent demographic developments in China—such as the earlier-than-expected population peak. Moreover, updated SSP projections are harmonised with the World Bank's short-term estimates (2020–2026) and apply SSP-specific growth rates thereafter[65]. These growth rates embed socioeconomic assumptions that may not align with the narratives used in our scenarios. Second, the UN's forecasts rely on standard demographic modelling techniques and are less influenced by assumptions about economic or governance trajectories, making them more independent our framework. Third, our scenario narratives incorporate assumptions about fertility and population dynamics that align closely with the range covered by the UN projections, ensuring consistency across our socioeconomic dimensions (see SI Table 8).

SI Table 7.Key population projection datasets issued from 2019 including China's projection

Name	Publish year	Spatial resolution	Temporal resolution	Scenario	Publisher
IIASA	2023	Country	2010-2100, by 5	SSP1-SSP5	Institute for Applied system analysis[63]
UN	2022	Country	1950-2100, by1	Estimates<br>Low fertility<br>Medium fertility<br>High fertility<br>Instant replacement-fertility<br>Momentum<br>Constant-mortality<br>No Change<br>Zero-migration	United Nations[66]
BNU	2022	Country and Provincial	2020-2100, by 1	SSP2	Beijing Normal University[67]
THU	2020	30 m	2010-2100, by1	SSP1-SSP5	Tsinghua University[66]
SEDAC	2020	1km	2010-2100, by 10	SSP1-SSP5	Socio economic data and application centre[68]
NUIST	2019	0.5 °	2010-2100, by 1	SSP1-SSP5	Nanjing University of information Science and Technology[69]
IHME	2019	Country	1950-2100， by 1	Reference, Slower, Faster, Fastest (female educational attainment)	Institute for Health Metrics and evaluation[70]

WCDE	2019	Country	1950-2100, by 5	SSP 1-Rapid development<br>SSP 2-medium<br>SSP 3-stalled development<br>SSP 2-Medium zero migration<br>SSP 2-Medium double migration	Wittgenstein Centre Data Explorer[71]

SI Table 8.Quantification of population changes in TIAM-UCL

Scenario	Scenario narratives regarding population	Fertility rate	Numerical assumptions of population (million)			
			**2030**	**2050**	**2080**	**2100**
**Great Wall**	**Accelerated decline: Economic Instability Undermines fertility intension**<br>Economic downturns lead to a deteriorating employment environment, undermining the confidence of younger generations. Additionally, concerns about healthcare, elderly care, education, and food safety make young people more cautious in decisions related to marriage and childbearing.	Low	1396.24	1221.77	769.46	494.14
**Calm Sea, Green Lights**	**Moderate decline: Rising Costs and Social Shifts Suppress Fertility**<br>With continued economic growth and rising per capita income, the costs of housing, education, healthcare, and childcare have significantly increased, making child-rearing more expensive. Furthermore, greater gender equality and women's empowerment have led to increased workforce participation, reducing societal pressure to have larger families.	Medium	1416.87	1316.95	978.55	771.30
**Red Sun**	**Alleviated decline: Social reforms and Economic Prosperity Drive Family Formation**<br>The complete abolition of population control policies, coupled with the implementation of hukou system reforms, could significantly enhance societal willingness to have children. Economic prosperity further boosts the confidence of younger generations in family formation.	High	1437.56	1413.10	1233.97	1154.04

4.4 Clean technology deployment

Clean technologies in the energy system are widely recognised as a cornerstone for mitigating climate change. In our scenario framework, clean technology development is differentiated both in narrative terms and in model quantification. We focus on two

representative technologies: solar photovoltaics (PV) and wind turbines. To project future technology costs, specifically capital costs and fixed operation and maintenance (O&M) costs, we apply an open-source, Python-based tool that enables exogenous cost projections with China-specific detail across scenarios, this tool is developed and firstly applied to MESSAGEix-GLOBIOM[72,73].

In this tool, one region can be defined as the "reference region", a technology leader whose cost trajectory other regions follow. For the reference region, future costs are projected using a "progress rate" based on Moore's Law, which assumes an exponential decline in costs over time. The progress rate varies by technology and scenario and is applied over the 2025–2100 horizon.

We adopt this approach for two main reasons. First, projecting technology costs across all sectors in an integrated assessment model (IAM) involves numerous assumptions and datasets, making it difficult to ensure consistency and transparency. This tool provides the flexibility to easily adjust technologies, regions, and scenarios. In our case, China is defined as the reference region, and we align the technology cost projections with our scenario-specific GDP assumptions, ensuring consistency between economic development and technological innovation. Second, many IAMs tend to overestimate energy transition costs by underestimating the pace of renewable cost reductions and deployment. This tool incorporates historical data from the IEA's World Energy Outlook (2023)[46] to derive base-year regional cost ratios, better capturing emerging market dynamics and regional variation—both critical for global modelling. Numerical assumptions generated from this tool are presented in SI Table 9.

SI Table 9.Quantification of Solar PV +Wind CAPEX of China in TIAM-UCL (Unit: 2005 USD/KW)

**High cost reduction ---Green Lights/Red Sun**				
	**2020**	**2030**	**2040**	**2050**
**Solar PV-Rooftop**	550	387	204	122
**Solar PV-Utility Scale**	495	348	183	109
**Offshore Wind**	1771	1465	1074	862
**Onshore Wind**	1181	977	716	575
**Medium cost reduction ---Calm Sea**				
	**2020**	**2030**	**2040**	**2050**
**Solar PV-Utility Scale**	550	444	311	241
**Solar PV-Rooftop**	495	400	280	217
**Offshore Wind**	1771	1541	1235	1059
**Onshore Wind**	1181	1028	823	706
**Low cost reduction ---Great Wall**				
	**2020**	**2030**	**2040**	**2050**
**Solar PV-Utility Scale**	550	517	468	437
**Solar PV-Rooftop**	495	465	421	393

**Offshore Wind**	1771	1663	1506	1406
**Onshore Wind**	1181	1109	1004	938

4.5 CCS deployment

The significance of CCS in the global transition to carbon neutrality is consistently highlighted in global integrated assessment models (IAMs), as well as in the IPCC's Assessment Reports, IEA's World Energy Outlook and Energy Technology Perspectives. Many studies have confirmed the critical role of carbon capture and storage (CCS) in enabling China to achieve its net-zero targets[29,74,75].

As of now, approximately 40 CCUS demonstration and pilot projects are either operational or under construction in China, collectively achieving about 3 million tonnes of CO_2 reductions per year[30]. Most of these projects remain small-scale, with capture capacities ranging from 10,000 to 100,000 tonnes annually. Only two exceed 500,000 tonnes. The main sectors involved are power generation, chemicals, iron and steel, and cement—with the power and chemical industries alone accounting for over 40% of total capacity.

This study adopts the government's official estimates from the China CCUS Roadmap Report (2021)[30] as the basis for our scenario assumptions, for three key reasons. First, given that most current CCUS projects are still in the demonstration phase and face financial viability challenges due to high capital costs and low or negative internal rates of return (IRR), government and state-owned enterprise involvement will continue to be the primary driver of near-term development. Therefore, projections from official sources are more credible and reflect realistic expectations. Second, since our scenarios are featured with policy granularity, government-derived estimates align more consistently with the narrative framework. Third, the China CCUS Roadmap Report provides a comprehensive, policy-informed estimate range across subsectors, synthesised from over 20 studies, and thus better captures the uncertainties surrounding future CCS deployment. Accordingly, we use the high-end estimates for Green Lights, the low-end for Great Wall, and the mid-range values for Red Sun and Calm Sea (see SI Table 10).

SI Table 10.Quantification of CCS deployment of China in TIAM-UCL ((units: Mt CO2/ year)

**High estimation ----Green Lights**						
	**2025**	**2030**	**2035**	**2040**	**2050**	**2060**
**Coal-fired plant**	6	20	100	500	500	500
**Gas power plant**	1	5	100	100	100	100
**Steel and iron**	1	5	20	30	70	110
**BECCS**	0.5	1	18	100	500	600
**DACCS**	0	0	1	15	100	300

Medium estimation ---Red Sun and Calm Sea						
	**2025**	**2030**	**2035**	**2040**	**2050**	**2060**
**Coal-fired plant**	6	20	100	350	350	350
**Gas power plant**	1	5	60	60	60	60
**Steel and iron**	1	3.5	15	25	60	100
**BECCS**	0.5	1	18	90	350	450
**DACCS**	0	0	1	15	75	250
**Low estimation ----Calm Sea**						
	**2025**	**2030**	**2035**	**2040**	**2050**	**2060**
**Coal-fired plant**	6	20	5	200	200	200
**Gas power plant**	1	5	20	20	20	20
**Steel and iron**	1	2-5	10	20	50	90
**BECCS**	0.5	1	18	80	200	300
**DACCS**	0	0	1	15	50	200

SI Section 5: Energy Service Demand in TIAM-UCL

This supplementary information outlines the energy service demand inputs used in TIAM-UCL, based on the scenario assumptions for China described in SI Section 4. TIAM-UCL represents final energy demand across four sectors—residential, commercial, transport, and industry—using a set of exogenously defined end-use energy service demands (ESDs). These demands are specified at the regional level and cover services such as residential space heating, domestic aviation, and iron and steel production (see SI Table 11 for the complete list).

SI Table 11.List of energy service demands in TIAM-UCL

Code	Service demand	Unit	Driver
ICH	Chemicals	PJ	PICH
IIS	Iron and Steel	Mt	PIIS
INF	Non-ferrous metals	Mt	PINF
INM	Non-Metals Mineral	PJ	PINM
ILP	Pulp and Paper	Mt	PILP
IOI	Other Industries	PJ	POI
NEO	Industrial and Other Non-Energy Uses	PJ	GDP
ONO	Other non-specified consumption	PJ	GDP
AGR	Agricultural demand	PJ	PAGR
CC1	Commercial Cooling - Region 1	PJ	PSER
CCK	Commercial Cooking	PJ	PSER
CH1	Commercial Space Heat - Region 1	PJ	PSER
CHW	Commercial Hot Water	PJ	PSER
CLA	Commercial Lighting	PJ	PSER
COE	Commercial Office Equipment	PJ	PSER
CRF	Commercial Refrigeration	PJ	PSER

RC1	Residential Cooling - Region 1	PJ	HOU/GDPPHOU*
RCD	Residential Clothes Drying	PJ	HOU/GDPPHOU*
RCW	Residential Clothes Washing	PJ	HOU/GDPPHOU*
RDW	Residential Dishwashing	PJ	HOU/GDPPHOU*
REA	Residential Other Electric	PJ	HOU/GDPPHOU*
RH1	Residential Space Heat - Region 1	PJ	HOU
RHW	Residential Hot Water	PJ	POP
RK1	Residential Cooking - Region 1	PJ	POP
RL1	Residential Lighting - Region 1	PJ	GDPP
RRF	Residential Refrigeration	PJ	HOU/GDPPHOU*
NEU	Non-Energy Uses	PJ	GDP
TAD	Domestic Aviation	PJ	GDP
TAI	International Aviation	PJ	GDP
TRB	Road Bus Demand	Bv-km	POP
TRC	Road Commercial Trucks Demand	Bv-km	GDP
TRE	Road Three Wheels Demand	Bv-km	POP
TRH	Road Heavy Trucks Demand	Bv-km	GDP
TRL	Road Light Vehicle Demand	Bv-km	GDP
TRM	Road Medium Trucks Demand	Bv-km	GDP
TRT	Road Auto Demand	Bv-km	GDPP
TRW	Road Two Wheels Demand	Bv-km	POP
TTF	Rail-Freight	PJ	GDP
TTP	Rail-Passengers	PJ	POP
TWD	Domestic Internal Navigation	PJ	GDP
TWI	International Navigation	PJ	GDP
*Driver is GDPPHOU for AFR, CHI, CSA, EEU, FSU, IND, MEA, MEX, ODA and SKO			

Each service demand is projected from 2005-2100 by relating it to a given demand driver and using the expression:

$$ESD_t = ESD_{t-1}\left(\frac{Driver_t}{Driver_{t-1}}\right)^{\propto_t}$$

Here α is a decoupling factor which is used to adjust the strength of the relationship between demand driver and ESD, thereby reflecting shifts in the wider socio-economic system. Drivers are listed in Table 11 and their code explained in Table 12. The ESDs ICH, IIS, INF, INM, ILP and AGR do not use a decoupling factor and are directly linked to their respective drivers.

SI Table 12.List of drivers for projecting energy service demands in TIAM-UCL

Driver	Description
PICH	Production of chemicals
PIIS	Production of iron and steel
PINF	Production of non-ferrous metals
PINM	Production of cement

PILP	Production of pulp and paper
PIOI	Production of other industries
PAGR	Production of agriculture (linked to calories consumed)
PSER	Services (directly linked to GDP growth)
HOU	Number of households
POP	Population
GDP	GDP
GDPP	GDP per capita
GDPPHOU	GDP per capita per household

In this analysis, China's key energy service demands such as heating, cooling, and iron and steel production, are modelled using scenario-specific socio-economic assumptions (see SI Section 4). The evolution of these demands across China's scenarios is shown in the SI Figure 4 below. For the rest of the world, energy service demands follow the SSP2 drivers.

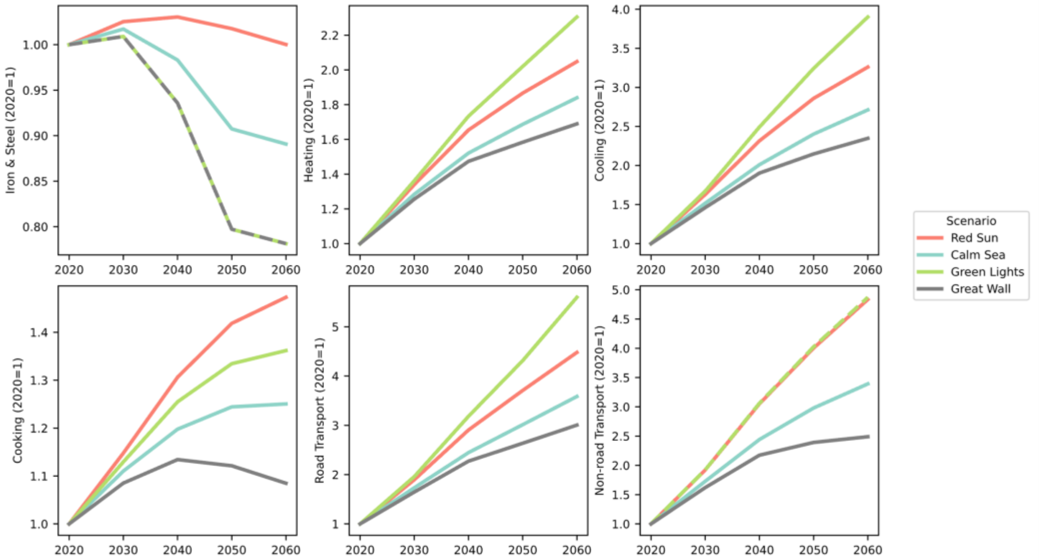

SI Figure 4.Projected growth in key energy service demands in China modelled in TIAM-UCL (relative to 2020)

SI Section 6: Sensitivity analysis

We identify two sensitivity dimensions in this study:

6.1 Credibility score weighting

The credibility score is based on a weighted average of three indicators, which may affect the policy cluster across all scenarios. We therefore test different weightings of the three indicators:

- $\omega_{gov}:\omega_{FYP}:\omega_{TNPI} = 3:2:5$
- $\omega_{gov}:\omega_{FYP}:\omega_{TNPI} = 2:3:5$
- $\omega_{gov}:\omega_{FYP}:\omega_{TNPI} = 3:3:4$
- $\omega_{gov}:\omega_{FYP}:\omega_{TNPI} = 1:1:1$

- $\omega_{gov}:\omega_{FYP}:\omega_{TNPI} = 4:4:2$

Results show that our clusters are largely robust across five different weighting combinations (SI Table 13). When the weight of the progress indicator is decreased relative to the two governance indicators, two targets show rating uncertainty:

- *By 2025, renewable energy will account for about 18% of primary energy consumption* (previously rated low in main case, now becomes medium).
- *By 2030, electricity will account for 65% of building energy consumption* (previously rated medium in main case, now becomes low).

As a result, we generate a sensitivity case of **Calm Sea** scenario, donated **CS_Low_TNPI**, which includes the first target but excludes the second. Since Green Lights includes all scenario clusters, it is unaffected in modelling practice. Sensitivity results for **CS_Low_TNPI** will be presented together with the sensitivity cases of GHG reduction in SI Section 6.2.

SI Table 13.Sensitivity of credibility assessment to weighting schemes

Numercial Target	Main case	W_gov:W_FYP:W_Prog=3:2:5	_FYP:W_Prog=2:3:5	W_gov:W_FYP:W_Prog=3:3:4	W_gov:W_FYP:W_Prog=1:1:1	W_gov:W_FYP:W_Prog=4:4:2
By 2025, energy consumption per unit of GDP will be lowered by 13.5% from the 2020 level.	MEDIUM	MEDIUM	MEDIUM	MEDIUM	MEDIUM	MEDIUM
By 2025, carbon dioxide (CO_2) emissions per unit of GDP will be lowered by 18% from the 2020 level.	MEDIUM	MEDIUM	MEDIUM	MEDIUM	MEDIUM	MEDIUM
By 2025, the share of non-fossil energy consumption will reach around 20%.	HIGH	HIGH	HIGH	HIGH	HIGH	HIGH
By 2025, the forest coverage rate will reach 24.1%, and the forest stock volume will rise to 18 billion cubic meters.	ACHIEVED	ACHIEVED	ACHIEVED	ACHIEVED	ACHIEVED	ACHIEVED
By 2025, electricity will account for about 30% of end-use energy consumption.	MEDIUM	MEDIUM	MEDIUM	MEDIUM	MEDIUM	MEDIUM
By 2025, total renewable energy consumption will reach about 1 billion tons of standard coal (~29,300 PJ).	MEDIUM	MEDIUM	MEDIUM	MEDIUM	MEDIUM	MEDIUM
By 2025, renewable energy will account for about 18% of primary energy consumption.	LOW	LOW	LOW	LOW	LOW	MEDIUM
By 2025, renewable energy will account for more than 50% of the incremental increase in primary energy consumption.	MEDIUM	MEDIUM	MEDIUM	MEDIUM	MEDIUM	MEDIUM
By 2025, the non-electric utilization of renewable energy (including geothermal heating, biomass heating, biomass fuels, and solar thermal utilization) will reach over 60 million tons of standard coal (~1,758 PJ).	MEDIUM	MEDIUM	MEDIUM	MEDIUM	MEDIUM	MEDIUM
By 2025, annual crude oil production will stabilize at around 200 million tons.	ACHIEVED	ACHIEVED	ACHIEVED	ACHIEVED	ACHIEVED	ACHIEVED
By 2025, annual natural gas production will exceed 230 billion cubic meters.	ACHIEVED	ACHIEVED	ACHIEVED	ACHIEVED	ACHIEVED	ACHIEVED
By 2025, 100,000–200,000 tons of hydrogen will be produced annually from renewable energy.	ACHIEVED	ACHIEVED	ACHIEVED	ACHIEVED	ACHIEVED	ACHIEVED
By 2025, total installed power generation capacity will reach approximately 3,000 GW.	ACHIEVED	ACHIEVED	ACHIEVED	ACHIEVED	ACHIEVED	ACHIEVED
By 2025, the proportion of non-fossil energy in power generation will reach about 39%.	MEDIUM	MEDIUM	MEDIUM	MEDIUM	MEDIUM	MEDIUM
By 2025, the operational installed capacity of nuclear power will reach approximately 70 GW.	ACHIEVED	ACHIEVED	ACHIEVED	ACHIEVED	ACHIEVED	ACHIEVED
By 2025, power generation from renewable energy will reach approximately 3,300 TWh.	ACHIEVED	ACHIEVED	ACHIEVED	ACHIEVED	ACHIEVED	ACHIEVED
By 2025, 33% of electricity will be generated from renewables.	ACHIEVED	ACHIEVED	ACHIEVED	ACHIEVED	ACHIEVED	ACHIEVED
By 2025, 18% of electricity will come from non-hydropower renewables.	ACHIEVED	ACHIEVED	ACHIEVED	ACHIEVED	ACHIEVED	ACHIEVED
By 2025, approximately 40 GW of additional hydropower capacity will be installed compared to 2020 (370 GW).	ACHIEVED	ACHIEVED	ACHIEVED	ACHIEVED	ACHIEVED	ACHIEVED
By 2025, CO_2 emissions per unit of industrial added value will be reduced by 18%.	LOW	LOW	LOW	LOW	LOW	LOW
By 2025, energy consumption per unit of industrial added value in large-scale industries will decrease by 13.5% compared to 2020.	LOW	LOW	LOW	LOW	LOW	LOW
By 2025, the proportion of EAF in iron and steel production will increase to 15%.	LOW	LOW	LOW	LOW	LOW	LOW
By 2025, recycled steel use will reach 320 million tons (updated to 300 million tons in the latest 2024 policy).	LOW	LOW	LOW	LOW	LOW	LOW
By 2025, domestic primary crude oil refining capacity will be kept below 1 billion metric tons (20 million b/d), and the utilization rate of production capacity for main products will rise to 80% or more.	ACHIEVED	ACHIEVED	ACHIEVED	ACHIEVED	ACHIEVED	ACHIEVED
By 2025, comprehensive energy consumption per ton of steel will be reduced by over 2% compared to 2020 (updated in 2024 to a 2% reduction compared to 2023).	LOW	LOW	LOW	LOW	LOW	LOW
By 2025, electricity will account for approximately 30% of industrial end-use energy consumption.	LOW	LOW	LOW	LOW	LOW	LOW
By 2025, the carbon intensity of road transportation will be reduced by 5% compared to 2020.	ACHIEVED	ACHIEVED	ACHIEVED	ACHIEVED	ACHIEVED	ACHIEVED
By 2025, the average electricity consumption of new passenger BEVs will be ≤12.0 kWh/100 km.	ACHIEVED	ACHIEVED	ACHIEVED	ACHIEVED	ACHIEVED	ACHIEVED
By 2025, the average fuel consumption of new passenger cars will be reduced to 4.0 liters/100 km.	ACHIEVED	ACHIEVED	ACHIEVED	ACHIEVED	ACHIEVED	ACHIEVED
By 2025, new energy buses will account for 72% of all surface public transport vehicles in urban areas.	ACHIEVED	ACHIEVED	ACHIEVED	ACHIEVED	ACHIEVED	ACHIEVED
By 2025, sales of new energy vehicles will account for about 20% of total new car sales.	ACHIEVED	ACHIEVED	ACHIEVED	ACHIEVED	ACHIEVED	ACHIEVED
By 2025, electricity consumption will account for over 55% of building energy consumption.	ACHIEVED	ACHIEVED	ACHIEVED	ACHIEVED	ACHIEVED	ACHIEVED
By 2025, renewable energy use in urban buildings will reach 8%.	MEDIUM	MEDIUM	MEDIUM	MEDIUM	MEDIUM	MEDIUM
By 2030, CO_2 emissions per unit of GDP will be reduced by over 65% from the 2005 level.	HIGH	HIGH	HIGH	HIGH	HIGH	HIGH
By 2030, the share of non-fossil fuels in primary energy consumption will reach around 25%.	HIGH	HIGH	HIGH	HIGH	HIGH	HIGH
By 2030, the forest coverage rate will reach about 25%, and the forest stock volume will reach 19 billion cubic meters.	ACHIEVED	ACHIEVED	ACHIEVED	ACHIEVED	ACHIEVED	ACHIEVED
By 2030, approximately 40 GW of additional hydropower capacity will be installed compared to 2025 (370 GW).	HIGH	HIGH	HIGH	HIGH	HIGH	HIGH
By 2030, the total installed capacity of wind and solar power will exceed 1,200 GW.	ACHIEVED	ACHIEVED	ACHIEVED	ACHIEVED	ACHIEVED	ACHIEVED
By 2030, around 40% of incremental vehicles will be fueled by new and clean energy.	HIGH	HIGH	HIGH	HIGH	HIGH	HIGH
By 2030, petroleum consumption for land transportation will peak before 2030.	HIGH	HIGH	HIGH	HIGH	HIGH	HIGH
By 2030, the average fuel consumption of new passenger cars will be reduced to 3.2 liters/100 km.	ACHIEVED	ACHIEVED	ACHIEVED	ACHIEVED	ACHIEVED	ACHIEVED
By 2030, 100% of public vehicles will be electrified.	HIGH	HIGH	HIGH	HIGH	HIGH	HIGH
By 2030, the proportion of EAF in iron and steel production will increase to over 20%.	LOW	LOW	LOW	LOW	LOW	LOW
By 2030, electricity will account for over 65% of building energy consumption.	MEDIUM	MEDIUM	MEDIUM	MEDIUM	MEDIUM	LOW
By 2035, the share of non-fossil fuels in total energy consumption will exceed 30%.	HIGH	HIGH	HIGH	HIGH	HIGH	HIGH
By 2035, the total installed capacity of wind and solar power generation will reach 3,600 GW, more than six times the 2020 level.	HIGH	HIGH	HIGH	HIGH	HIGH	HIGH
By 2060, the share of non-fossil fuels in energy consumption will exceed 80%.	LOW	LOW	LOW	LOW	LOW	LOW

6.2 China’s GHG emissions reduction target by 2035

At the United Nations General Assembly on 25 September 2025**,** President Xi announced China’s new 2035 NDC targets in a video address, outlining three core commitments:

Reduce economy-wide net GHG emissions by 7% to 10% below peak levels by 2035.
Increase the share of non-fossil energy in total energy consumption to over 30% by 2035.
Expand the installed capacity of wind + solar to 3,600 GW by 2035.

The targets on non-fossil energy share and wind and solar installed capacity are extensions of existing pledges. We integrated these into our policy credibility assessment framework, where they are rated as highly credible and implemented starting from the Red Sun scenario. By contrast, the GHG emissions target is China's first explicit absolute emissions reduction pledge. As there is no historical progress against which to benchmark it, we treat this as an additional policy sensitivity to our main case. Specifically, we impose this target in the Red Sun, Calm Sea, and Green Lights scenarios (but not in Great Wall, which represents a pathway where only existing targets are achieved), and test both a 7% and a 10% reduction. The reductions are measured relative to the emissions peak: 2025 for Green Lights, 2030 for Calm Sea, and for Red Sun (which has no explicit peak target) relative to 2030, to gauge its impact.

SI Figure 5 compares Chinese energy transition metrics across sensitivity cases, illustrating how they different from the main case. The colours represent the scenario families: Great Wall (grey), Red Sun (red), Calm Sea (blue), and Green Lights (green). Circles indicate the main case presented in the manuscript, while triangles and inverted triangles correspond to additional 7% and 10% GHG reduction targets by 2035, respectively, imposed on top of the main case. Diamonds represent the CS_Low_TNPI sensitivity. Results show that our findings of China's energy sensitivity across scenarios are largely robust to weighting scheme sensitivities.

SI Figure 5. (a) shows that coal generation declines sharply across all cases, while solar and wind generation rises substantially, consistent with the main case. Notably, the Red Sun and Calm Sea cases show that achieving the 2035 GHG reduction targets accelerates the phaseout of coal generation (an additional ~1,000 TWh annual reduction in 2040 compared with the main case), with hydropower providing a short-term offset before further solar and wind expansion. SI Figure 5. (b) also indicates that achieving the 2035 GHG 10% reduction target requires an additional ~10 EJ annual reduction in coal consumption in 2040 compared with the main case, which does not include a GHG reduction target.

SI Figure 6 illustrates the global implications of the sensitivity cases across our scenario families for cumulative emissions (Figure 6.(a)) and temperature outcomes (Figure 6.(b)). Including China's 2035 GHG emissions targets further reduces global cumulative CO_2 emissions by 2050 and 2100 but does not significantly alter the overall emission trends

across scenarios, nor does it challenge our findings regarding the differences driven by China's policy delivery. Achieving the additional 2035 GHG reduction targets could lower global cumulative CO_2 emissions by approximately 4–6 Gt in the 7% reduction case and 6–10 Gt in the 10% reduction case (see SI Table 14).

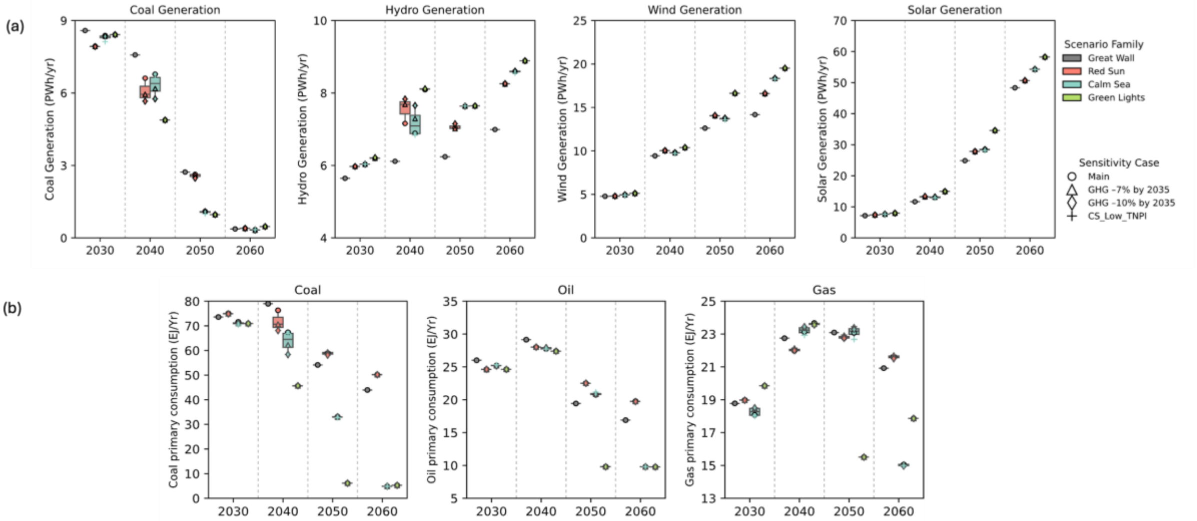

SI Figure 5.Chinese energy transition metrics across sensitivity cases (a) Power generation by key source: coal, hydro, wind, and solar (b) Fossil fuel consumption: coal, oil and gas. Box plots summarise outcomes across modelled China scenario realisations with different sensitivities (n = 22 per year). Centre lines indicate medians, boxes show the interquartile range (25th–75th percentiles), and whiskers extend to 1.5× the interquartile range. Individual points represent results from each scenario realisation; colours denote scenario families (Great Wall, Red Sun, Calm Sea and Green Lights), and marker shapes indicate sensitivity cases.

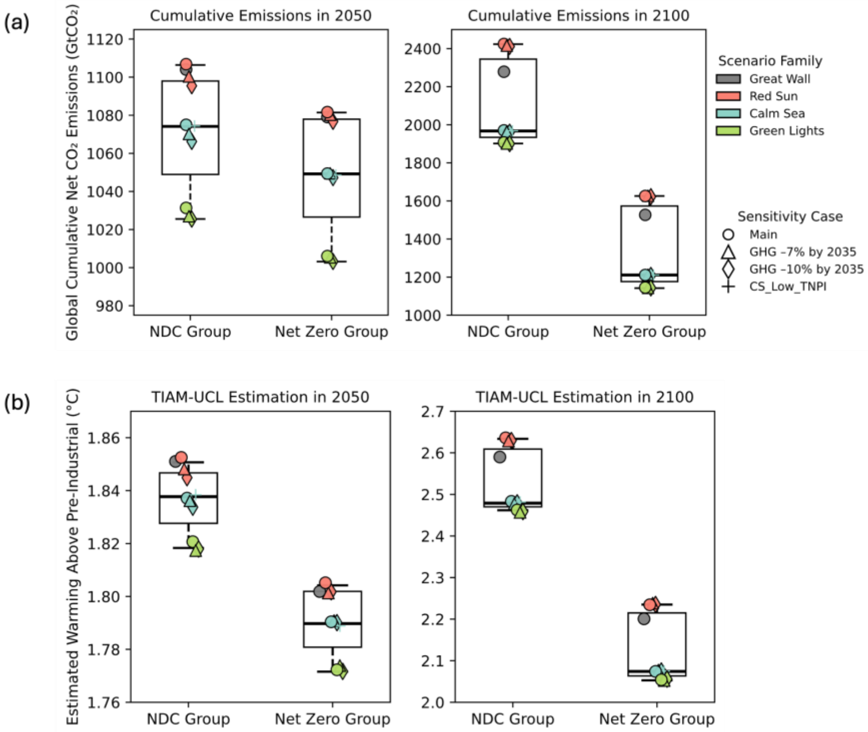

SI Figure 6.Global emissions and temperature metrics across sensitivity cases (a) Global cumulative emissions in 2050 and 2100, and (b) TIAM temperature increases estimation in 2050 and 2100. Box plots summarise

outcomes across modelled China scenario realisations within each global policy framework (n = 11 per group). Centre lines indicate medians, boxes show the interquartile range (25th–75th percentiles), and whiskers extend to 1.5× the interquartile range. Individual points represent results for each scenario realisation; colours denote scenario families (Great Wall, Red Sun, Calm Sea and Green Lights) and marker shapes indicate sensitivity cases. Horizontal offsets and small vertical jitter are applied for visual clarity only and do not represent additional variability.

SI Table 14.Additional global cumulative emission reductions in 2050 and 2100 from China's 2035 GHG emissions targets

	Cumulative emissions reduction relative to the corresponding main case (Mt CO_2)	
	2050	2100
RS_7% GHG reduction by 2035	6188	6655
RS_10% GHG reduction by 2035	10721	11504
CS_7% GHG reduction by 2035	5158	5085
CS_10% GHG reduction by 2035	8938	8798
GL_7% GHG reduction by 2035	4032	4005
GL_10% GHG reduction by 2035	5933	5931

Supplementary reference

1. Markard, J., Raven, R. & Truffer, B. Sustainability transitions: An emerging field of research and its prospects. *Research Policy* **41**, 955–967 (2012).
2. Nemet, G. F., Jakob, M., Steckel, J. C. & Edenhofer, O. Addressing policy credibility problems for low-carbon investment. *Global Environmental Change* **42**, 47–57 (2017).
3. Rogelj, J. *et al.* Credibility gap in net-zero climate targets leaves world at high risk. *Science* **380**, 1014–1016 (2023).
4. Teng, F. Ambitious and credible pledges. *Nat. Clim. Chang.* **12**, 779–780 (2022).
5. Victor, D. G., Lumkowsky, M. & Dannenberg, A. Determining the credibility of commitments in international climate policy. *Nat. Clim. Chang.* **12**, 793–800 (2022).
6. Brunner, S., Flachsland, C. & Marschinski, R. Credible commitment in carbon policy. *Climate Policy* **12**, 255–271 (2012).
7. Jacobs, A. M. Policy Making for the Long Term in Advanced Democracies. *Annual Review of Political Science* **19**, 433–454 (2016).

8. Rogge, K. S. & Reichardt, K. Policy mixes for sustainability transitions: An extended concept and framework for analysis. *Research Policy* **45**, 1620–1635 (2016).
9. Zhu, M. The Rule of Climate Policy: How Do Chinese Judges Contribute to Climate Governance without Climate Law? *Transnational Environmental Law* (2021).
10. Oxford Institute of Enegy Studies. Appendix A: Government Structure. *Guide to Chinese Climate Policy* https://chineseclimatepolicy.oxfordenergy.org/book-content/appendicies/government-structure/ (2021).
11. Xu, S. China's climate governance for carbon neutrality: regulatory gaps and the ways forward. *Humanit Soc Sci Commun* **10**, 1–10 (2023).
12. Teng, F. & Wang, P. The evolution of climate governance in China: drivers, features, and effectiveness. *Environmental Politics* **30**, 141–161 (2021).
13. Ran, R. Perverse Incentive Structure and Policy Implementation Gap in China's Local Environmental Politics. *Journal of Environmental Policy & Planning* **15**, 17–39 (2013).
14. Chen, H., Feng, L. & Sun, X. Beyond central-local relations: the introduction of a new perspective on China's environmental governance model. *Humanit Soc Sci Commun* **11**, 1–12 (2024).
15. Wiedenbach, A. Divergent Central and Local Government Interests May Impede Implementation of China's Climate Targets. *Climate Scorecard* https://www.climatescorecard.org/2021/08/divergent-central-and-local-government-interests-may-impede-implementation-of-chinas-climate-targets/ (2021).
16. Dai, A., Gordon, J. & Zhu, Ri. Looking back and looking ahead: AN ANALYSIS OF PROVINCIAL TH FIVE-YEAR PLANS (2021-2025). (2024).

17. Liu, H. What does China's 14th 'five year plan' mean for climate change? *Carbon Brief* https://www.carbonbrief.org/qa-what-does-chinas-14th-five-year-plan-mean-for-climate-change/ (2021).
18. Ministry of Ecology and Environment. Progress on the Implementation of China's Nationally Determined Contributions (2022). (2022).
19. IEA. China: CO_2 emissions. *IEA* https://www.iea.org/countries/china/emissions (2023).
20. McConaughey, M. *Green Lights*. (Crown, 2020).
21. The World Bank. China Country Climate and Development Report. https://www.worldbank.org/en/country/china/publication/china-country-climate-and-development-report (2023).
22. State Council. China's new 'dual circulation' development paradigm. https://english.www.gov.cn/news/topnews/202103/28/content_WS60604adbc6d0719374afba4a.html (2021).
23. State Council. Strides made in advancing Beautiful China Initiative: official. https://english.www.gov.cn/news/topnews/202210/21/content_WS63526392c6d0a757729e17e0.html (2022).
24. State Council. China pushes 'dual circulation' to power growth in new development stage. https://english.www.gov.cn/news/topnews/202103/10/content_WS60486347c6d0719374afa854.html (2021).
25. Central Committee of the Communist Party & State Council. Opinions of the Central Committee of the Communist Party of China and the State Council on Comprehensively Promoting the Construction of a Beautiful China 中共中央 国务院关于全面推进美丽

中国建设的意见_中央有关文件_中国政府网. https://www.gov.cn/zhengce/202401/content_6925405.htm (2024).

26. State Council. Implementation stressed for manufacturing vision. https://english.www.gov.cn/policies/latest_releases/2015/05/19/content_281475110703534.htm.

27. Conroy, G. How 'Made in China 2025' helped supercharge scientific development in China's cities. *Nature* d41586-024-03522-y (2024) doi:10.1038/d41586-024-03522-y.

28. Arendse, H. China's New Quality Productive Forces: An Explainer. *China Briefing News* https://www.china-briefing.com/news/chinas-new-quality-productive-forces-an-explainer/ (2024).

29. Wang, P. *et al.* CCUS development in China and forecast its contribution to emission reduction. *Sci Rep* **13**, 17811 (2023).

30. Ministry of Ecology and Environment. China Carbon Dioxide Capture, Utilization and Storage (CCUS) Annual Report (2021)-China CCUS Pathway Research (2021) 中国二氧化碳捕集利用与封 存 (CCUS) 年度报告. (2021).

31. State Council. China's home appliance sales surge in 2024 under trade-in scheme. https://english.www.gov.cn/archive/statistics/202501/19/content_WS678c38fac6d0868f4e8eef62.html.

32. State Council. Consumption vouchers issued to boost spending in China. https://english.www.gov.cn/news/202409/26/content_WS66f51bc1c6d0868f4e8eb4af.html.

33. State Council. China’s property market shows stronger signs of recovery. https://english.www.gov.cn/archive/statistics/202412/16/content_WS6760192fc6d0868f4e8ee041.html (2024).
34. State Council. New urbanization implementation plan approved. https://english.www.gov.cn/policies/latestreleases/202206/07/content_WS629f170ac6d02e533532bcf7.html (2022).
35. State Council. China unveils new measures to stabilize housing market. https://english.www.gov.cn/news/202410/17/content_WS67111067c6d0868f4e8ebfd0.html (2024).
36. Gao, C., Chang, S. & Wang, Y. From global to national: The role of urban agglomerations in China’s new development paradigm. *PLoS ONE* **19**, e0305594 (2024).
37. Wang, S., Yang, C., Hou, D. & Dai, L. How do urban agglomerations drive economic development? A policy implementation and spatial effects perspective. *Economic Analysis and Policy* **80**, 1224–1238 (2023).
38. UN. World Population Prospects 2024 | Population Division. https://www.un.org/development/desa/pd/world-population-prospects-2024 (2024).
39. NDRC. Implementation Plan for New Urbanization in the 14th Five-Year Plan. (2022).
40. Central Committee of the Chinese Communist Party & State Council. Decision of the Central Committee of the Chinese Communist Party and of the State Council 中共中央国务院关于优化生育政策促进人口长期均衡发展的决定_中央有关文件_中国政府网. https://www.gov.cn/zhengce/2021-07/20/content_5626190.htm.
41. Arendse, H. Urbanization in China - New Action Plan to Facilitate Urban Migration. https://www.china-briefing.com/news/urbanization-in-china-action-plan-migration/.

42. Interesse, G. China Tourism in 2023: Outlook, Trends and Opportunities. https://www.china-briefing.com/news/chinas-tourism-in-2023-outlook-trends-and-opportunities/.
43. You, X. The ‘new three’: How China came to lead solar, battery and EV manufacturing. https://dialogue.earth/en/business/new-three-china-solar-cell-lithium-battery-ev/ (2023).
44. Myllyvirta, L. Analysis: China’s emissions set to fall in 2024 after record growth in clean energy. *Carbon Brief* https://www.carbonbrief.org/analysis-chinas-emissions-set-to-fall-in-2024-after-record-growth-in-clean-energy/ (2023).
45. IEA. World Energy Outlook 2024. https://www.iea.org/reports/world-energy-outlook-2024 (2024).
46. IEA. World Energy Outlook 2023 – Analysis. *IEA* https://www.iea.org/reports/world-energy-outlook-2023 (2023).
47. The World Bank. China Overview: Development news, research, data. https://www.worldbank.org/en/country/china/overview (2024).
48. Rogoff, K. & Yang, Y. Rethinking China’s growth. *Economic Policy* **39**, 517–548 (2024).
49. IMF. China’s Real Estate Challenge. https://www.imf.org/en/Publications/fandd/issues/2024/12/chinas-real-estate-challenge-kenneth-rogoff (2024).
50. Wang, Y., Zhou, Y., Yu, X. & Liu, X. Is domestic consumption dragged down by real estate sector?—Evidence from Chinese household wealth. *International Review of Financial Analysis* **75**, 101749 (2021).

51. Ma, L. & Christensen, T. Government Trust, Social Trust, and Citizens' Risk Concerns: Evidence from Crisis Management in China. *Public Performance & Management Review* **42**, 383–404 (2019).
52. China off track on emissions goals as energy demand offsets renewables push, researchers say. *Reuters* (2024).
53. The Straits Times. China at risk of missing 2025 climate targets, needs steep CO2 cuts: Study. *Carbon Brief* https://www.carbonbrief.org/daily-brief/china-at-risk-of-missing-2025-climate-targets-needs-steep-co2-cuts-study/ (2024).
54. Naughton, B. *The Chinese Economy: Transitions and Growth*. (MIT Press, Cambridge, Mass, 2007).
55. Naughton, B. J. The Chinese Economy: Adaptation and Growth, second edition. *MIT Press Books* **1**, (2018).
56. Knight, J. & Ding, S. *China's Remarkable Economic Growth*. (Oxford University Press, 2012). doi:10.1093/acprof:oso/9780199698691.001.0001.
57. ICCSD. *China's Long-Term Low-Carbon Development Strategies and Pathways: Comprehensive Report*. (Springer Singapore, Singapore, 2022). doi:10.1007/978-981-16-2524-4.
58. CNBC. China 2024 GDP forecasts by JPMorgan, Goldman, Citi, Morgan Stanley. https://www.cnbc.com/2024/01/17/china-2024-gdp-forecasts-by-jpmorgan-goldman-citi-morgan-stanley.html (2024).
59. IMF. *World Economic Outlook Update, January 2024: Moderating Inflation and Steady Growth Open Path to Soft Landing*. (2024).
60. JCER. China's Growth Rate Below 3% after 2029 | Japan Center for Economic Research. https://www.jcer.or.jp/english/chinas-growth-rate-below-3-after-2029 (2023).

61. Carnegie. Can China's Long-Term Growth Rate Exceed 2–3 Percent? - Carnegie Endowment for International Peace. https://carnegieendowment.org/chinafinancialmarkets/89466 (2023).
62. O'Neill, B. C. *et al.* The roads ahead: Narratives for shared socioeconomic pathways describing world futures in the 21st century. *Global Environmental Change* **42**, 169–180 (2017).
63. IIASA. Shared Socioeconomic Pathways Scenario Database (SSP) 3.0 realse. *IIASA - International Institute for Applied Systems Analysis* http://iiasa.ac.at/models-tools-data/ssp (2024).
64. National Bureau of Statistics of China. Main Data of the Seventh National Population Census. https://www.stats.gov.cn/english/PressRelease/202105/t20210510_1817185.html (2021).
65. Koch, J. & Leimbach, M. SSP economic growth projections: Major changes of key drivers in integrated assessment modelling. *Ecological Economics* **206**, 107751 (2023).
66. Chen, Y. *et al.* Provincial and gridded population projection for China under shared socioeconomic pathways from 2010 to 2100. *Sci Data* **7**, 83 (2020).
67. Dai, K., Shen, S. & Cheng, C. Evaluation and analysis of the projected population of China. *Sci Rep* **12**, 3644 (2022).
68. Gao, A. J. Downscaling Global Spatial Population Projections from 1/8-degree to 1-km Grid Cells. https://opensky.ucar.edu/islandora/object/technotes%3A553 (2017).
69. Huang, J. *et al.* Effect of Fertility Policy Changes on the Population Structure and Economy of China: From the Perspective of the Shared Socioeconomic Pathways. *Earth's Future* **7**, 250–265 (2019).

70. IHME. Homepage | Institute for Health Metrics and Evaluation. https://www.healthdata.org/ (2019).
71. WCDE. Wittgenstein Centre Human Capital Data Explorer. https://dataexplorer.wittgensteincentre.org/wcde-v2/ (2019).
72. Meng, M., Krey, V., Way, R., Kim, J. & Woo, J.-H. A generalized tool for projecting investment and fixed O&M costs across technologies, scenarios, and regions. in (2024).
73. Way, R., Ives, M. C., Mealy, P. & Farmer, J. D. Empirically grounded technology forecasts and the energy transition. *Joule* **6**, 2057–2082 (2022).
74. IEA. An energy sector roadmap to carbon neutrality in China. https://www.iea.org/reports/an-energy-sector-roadmap-to-carbon-neutrality-in-china (2021).
75. Chen, S., Liu, J., Zhang, Q., Teng, F. & McLellan, B. C. A critical review on deployment planning and risk analysis of carbon capture, utilization, and storage (CCUS) toward carbon neutrality. *Renewable and Sustainable Energy Reviews* **167**, 112537 (2022).
