# Supplementary material for: Global implications of uncertainty in China’s climate policy delivery
Source: Nat Commun. 2026 Mar 6;17:3544. doi: 10.1038/s41467-026-70400-8 (PMC13087136; doi:10.1038/s41467-026-70400-8)
Supplement: Supplementary file 2 — Reporting Summary [file 41467_2026_70400_MOESM2_ESM.pdf]

nature portfolio

Corresponding author(s): Dan Zhang

Last updated by author(s): Jan 24, 2026

Reporting Summary

Nature Portfolio wishes to improve the reproducibility of the work that we publish. This form provides structure for consistency and transparency in reporting. For further information on Nature Portfolio policies, see our Editorial Policies and the Editorial Policy Checklist.

Statistics

For all statistical analyses, confirm that the following items are present in the figure legend, table legend, main text, or Methods section.

n/a	Confirmed	
☐	☒	The exact sample size (n) for each experimental group/condition, given as a discrete number and unit of measurement
☒	☐	A statement on whether measurements were taken from distinct samples or whether the same sample was measured repeatedly
☒	☐	The statistical test(s) used AND whether they are one- or two-sided<br>*Only common tests should be described solely by name; describe more complex techniques in the Methods section.*
☒	☐	A description of all covariates tested
☒	☐	A description of any assumptions or corrections, such as tests of normality and adjustment for multiple comparisons
☐	☒	A full description of the statistical parameters including central tendency (e.g. means) or other basic estimates (e.g. regression coefficient) AND variation (e.g. standard deviation) or associated estimates of uncertainty (e.g. confidence intervals)
☒	☐	For null hypothesis testing, the test statistic (e.g. F, t, r) with confidence intervals, effect sizes, degrees of freedom and P value noted<br>*Give P values as exact values whenever suitable.*
☒	☐	For Bayesian analysis, information on the choice of priors and Markov chain Monte Carlo settings
☒	☐	For hierarchical and complex designs, identification of the appropriate level for tests and full reporting of outcomes
☒	☐	Estimates of effect sizes (e.g. Cohen's d, Pearson's r), indicating how they were calculated

Our web collection on statistics for biologists contains articles on many of the points above.

Software and code

Policy information about availability of computer code

Data collection	No software was used.
Data analysis	The TIAM-UCL model version used for this analysis was 4.1.2, and was run using TIMES code 4.5.6 with GAMS 38.<br>The model solver used was CPLEX 20.1.0.1. The underlying code (mathematical equations) for the model is available via GitHub (https://github.com/etsap-TIMES/TIMES_model).

For manuscripts utilizing custom algorithms or software that are central to the research but not yet described in published literature, software must be made available to editors and reviewers. We strongly encourage code deposition in a community repository (e.g. GitHub). See the Nature Portfolio guidelines for submitting code & software for further information.

Data

Policy information about availability of data

All manuscripts must include a data availability statement. This statement should provide the following information, where applicable:

- Accession codes, unique identifiers, or web links for publicly available datasets
- A description of any restrictions on data availability
- For clinical datasets or third party data, please ensure that the statement adheres to our policy

The processed data underlying the figures and analyses presented in this study, including source data for the policy credibility assessment, net-zero assumptions, and figure generation, have been deposited in the Zenodo repository (https://doi.org/10.5281/zenodo.18378439). The TIAM-UCL model outputs and scenario input files used in the analysis are available in the same repository.

Research involving human participants, their data, or biological material

Policy information about studies with human participants or human data. See also policy information about sex, gender (identity/presentation), and sexual orientation and race, ethnicity and racism.

Reporting on sex and gender	Not applicable, as we did not involve human participants
Reporting on race, ethnicity, or other socially relevant groupings	Not applicable, as we did not involve human participants
Population characteristics	Not applicable, as we did not involve human participants
Recruitment	Not applicable, as we did not involve human participants
Ethics oversight	Not applicable, as we did not involve human participants

Note that full information on the approval of the study protocol must also be provided in the manuscript.

Field-specific reporting

Please select the one below that is the best fit for your research. If you are not sure, read the appropriate sections before making your selection.

☐ Life sciences ☒ Behavioural & social sciences ☐ Ecological, evolutionary & environmental sciences

For a reference copy of the document with all sections, see nature.com/documents/nr-reporting-summary-flat.pdf

Behavioural & social sciences study design

All studies must disclose on these points even when the disclosure is negative.

Study description	This study conducts a document-based policy credibility assessment of China's national climate and energy policies. Using a structured review of policy documents issued between 2020 and September 2025, numerical policy targets are extracted, filtered for model applicability, and assessed using a quantitative credibility scoring framework. The resulting policy credibility scenarios are embedded within an integrated assessment model to evaluate implications for emissions and global climate outcomes.<br>We have chosen this category in this form as we felt our disciplinary background of economic and engineering analysis best fit with social sciences.
Research sample	The research sample consists of national-level climate and energy policy targets extracted from official Chinese government policy documents, including China's updated 2035 Nationally Determined Contribution (NDC), the "1+N" climate policy framework, the 14th Five-Year Plan, and sectoral energy and emissions policies. In total, 292 targets from 58 policy documents were identified, of which 47 quantifiable targets were retained for the credibility assessment.
Sampling strategy	A purposive document-based sampling strategy was applied, focusing on national-level policies relevant to China's climate mitigation and energy transition. Policies were selected to ensure comprehensive coverage of major government planning frameworks and sectoral instruments over the study period.
Data collection	Policy documents were identified through official Chinese government sources and supplemented using international policy databases, including the International Energy Agency and Climate Action Tracker. All numerical targets were extracted using a standardized data-collection template, recording policy source, sector, metric, baseline year, target year, and quantitative value. Data extraction, filtering criteria, and full documentation are provided in the Supplementary Information.
Timing	Policy collection and target extraction were conducted between 2023 and September 2025. Progress against targets was reviewed using publicly available information up to November 2025.
Data exclusions	Exclusion criteria were defined a priori based on model representability and target measurability. Targets were excluded if they related to technologies, commodities, pollutants, financial instruments, or resource flows not represented in the integrated assessment model, or if they lacked clear quantitative definitions. Detailed exclusion rationales and examples are provided in

	Supplementary Information Table 2.
Non-participation	Not applicable. This study does not involve human participants, recruitment, or surveys.
Randomization	Not applicable. This study is an observational document-based analysis and does not involve experimental interventions or random assignment.

Reporting for specific materials, systems and methods

We require information from authors about some types of materials, experimental systems and methods used in many studies. Here, indicate whether each material, system or method listed is relevant to your study. If you are not sure if a list item applies to your research, read the appropriate section before selecting a response.

Materials & experimental systems

n/a	Involved in the study	
☒	☐	Antibodies
☒	☐	Eukaryotic cell lines
☒	☐	Palaeontology and archaeology
☒	☐	Animals and other organisms
☒	☐	Clinical data
☒	☐	Dual use research of concern
☒	☐	Plants

Methods

n/a	Involved in the study	
☒	☐	ChIP-seq
☒	☐	Flow cytometry
☒	☐	MRI-based neuroimaging

Plants

Seed stocks	Not applicable, as we did not involve plants
Novel plant genotypes	Not applicable, as we did not involve plants
Authentication	Not applicable, as we did not involve plants
